# Unveiling the Growth Mechanism of Ordered‐Phase within Multimetallic Nanoplates

**DOI:** 10.1002/advs.202309163

**Published:** 2024-02-29

**Authors:** Azhar Mahmood, Dequan He, Chuhao Liu, Shamraiz Hussain Talib, Bolin Zhao, Tianren Liu, Ying He, Lijuan Chen, Dongxue Han, Li Niu

**Affiliations:** ^1^ Center for Advanced Analytical Science, Guangzhou Key Laboratory of Sensing Materials and Devices, Guangdong Engineering Technology Research Center for Photoelectric Sensing Materials and Devices, School of Chemistry and Chemical Engineering Guangzhou University Guangzhou 510006 P. R. China; ^2^ Department of Chemistry Tsinghua University Beijing 100084 P. R. China; ^3^ College of Chemistry and Molecular Engineering Peking University Beijing 100871 P. R. China; ^4^ Advanced Materials Chemistry Centre Khalifa University of Science and Technology Abu Dhabi 127788 UAE; ^5^ School of Chemical Engineering and Technology Sun Yat‐sen University Zhuhai 519082 P. R. China

**Keywords:** catalysis, intermetallic, metal nanocrystals, ordered‐phase, strain effect

## Abstract

Tuning the crystal phase of alloy nanocrystals (NCs) offers an alternative way to improve their electrocatalytic performance, but, how heterometals diffuse and form ordered‐phase remains unclear. Herein, for the first time, the mechanism for forming tetrametallic ordered‐phase nanoplates (NPLs) is unraveled. The observations reveal that the intermetallic ordered‐phase nucleates through crystallinity alteration of the seeds and then propagates by reentrant grooves. Notably, the reentrant grooves act as intermediate NCs for ordered‐phase, eventually forming intermetallic PdCuIrCo NPLs. These NPLs substantially outperform for oxygen evolution reaction (221 mV at 10 mA cm^−2^) and hydrogen evolution reaction (19 mV at 10 mA cm^−2^) compared to commercial Ir/C and Pd/C catalysts in acidic media. For OER at 1.53 V versus RHE, the PdCuIrCo/C exhibits an enhanced mass activity of 9.8 A mg^−1^_Pd+Ir_ (about ten times higher) than Ir/C. For HER at ‐0. 2 V versus RHE, PdCuIrCo/C shows a remarkable mass activity of 1.06 A mg^−1^_Pd+Ir_, which is three‐fold relative to Pd/C. These improvements can be ascribed to the intermetallic ordered‐structure with high‐valence Ir sites and tensile‐strain. This approach enabled the realization of a previously unobserved mechanism for ordered‐phase NCs. Therefore, this strategy of making ordered‐phase NPLs can be used in diverse heterogeneous catalysis.

## Graphical abstract

By the utilization of crystallinity of the seeds and intermediate nanocrystals, ordered phase PdCuIr and PdCuIrCo nanoplates are obtained. These nanoplates outperform for oxygen evolution reaction and hydrogen evolution reaction compared to Ir/C and Pd/C catalysts in acidic media.

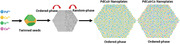

## 1 Introduction

Precise control over the crystal‐phase of metal nanocrystals (NCs) has made these materials of great interest for catalysis applications.^[^ ^1^ ^]^ One effective strategy to enhance the activity of metal NCs is engineering their structure with ordered‐phase.^[^ ^2^ ^]^ The ordered atomic arrangement in the ordered structures provoke electronic effects and intensified strain that influences the position of the d‐band center relative to the Fermi level, resulting in their superior catalytic performances.^[^ ^1^, ^3^ ^]^ Moreover, ordered structures exhibit lower heat of formation, which enhances their resistance to corrosion.^[^ ^4^, ^5^, ^6^ ^]^ For example, palladium (Pd) based intermetallic NCs show better activity and stability for oxygen evolution reaction (OER) and hydrogen evolution reaction (HER), compared with their analogous random alloy.^[^ ^7^, ^8^ ^]^ To date, several studies have been conducted to investigate the formation of ordered‐phase NCs. However, the state‐of the‐art studies for the formation of ordered‐phase NCs are mostly confined to bimetallic systems.^[^ ^4^, ^9^, ^10^ ^]^ Because, with the increment of the number of components in the alloy, their high mixing entropy could hinders the generation of monodisperse intermetallic compounds, which is an obstacle to the development of advanced intermetallic systems and it is a considerable challenge to control phase structures of intermetallic compounds precisely.^[^ ^11^ ^]^

Another reason that hinders the formation of multimetallic (tri‐ or tetrametallic) ordered‐phase NCs is the different reduction rate of each metal, which makes the reaction environment complex and the main question was remains as how to get control over the nucleation and formation process of multimetallic NCs to form ordered‐phase.^[^ ^1^, ^11^ ^]^ Moreover, the detailed mechanisms promoting the formation of intermetallic NCs are largely unknown because of the complex growth and ordering processes.^[^ ^4^, ^6^ ^]^ The experimental research and practical applications of intermetallic NCs are severely limited for the above reasons.^[^ ^9^ ^]^ Therefore, experimentally exploring the mechanisms and nanoscopic intermediate stages for the formation of intermetallic NCs is challenging. A more significant challenge is finding how the intermetallic phase nucleates and grows within tri‐ or tetrametallic components system.

In this study, for the first time, we address this challenge by combining the concepts of crystal symmetry and reentrant grooves with a simple solvothermal method. More specifically, we examined how the crystallinity alteration of the seeds lead to concave and convex edges and how the preferential growth at the edges (reentrant grooves) eventually leads to the formation of ordered intermetallic PdCuIr and PdCuIrCo nanoplates (NPLs). Geometric‐phase analysis demonstrates that the ordered‐phase can induce tensile‐strain in NPLs, and X‐ray absorption spectroscopy discloses that the oxidized states of Ir in ordered‐phase NPLs significantly impact the electronic structure. As a result, in the electrochemical water splitting, ordered‐phase multimetallic NPLs show an ultrahigh mass activity and substantially enhanced stability representing great superiority in intrinsic activity and durability toward water electrolysis under acidic conditions. Theoretical calculations combined with the surface property of ordered‐phase NPLs attributed the high water splitting performance to chiral atomic arrangements with high‐valence Ir sites. This effort will pay off significantly, as the best is yet to come in practical fuel cell applications.

## 2 Results and Discussion

**Figure 1:**
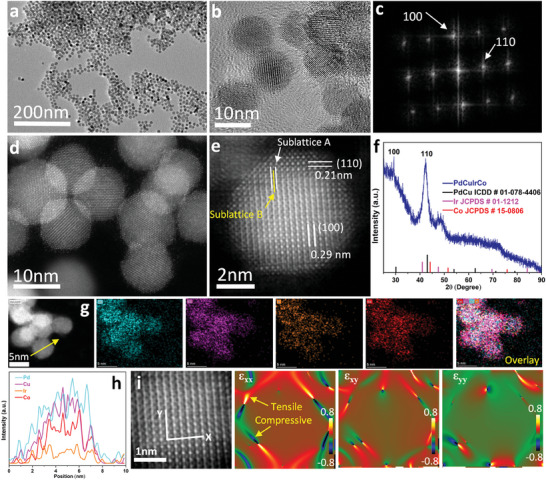
Structural and surface strain characterizations of the PdCuIrCo nanoplates: a) TEM image, b) HRTEM image, c) FFT Pattern, d) HAADF‐STEM image, e) Abreaction‐corrected HAADF‐STEM image, f) XRD Pattern, g) HAADF‐STEM image and the corresponding EELS elemental mapping, h) line‐scanning profiles across the yellow arrow shown in the image of insets (g) and (i) maps of the in‐plan strain tensors εxx, εyy, εxy, processed via GPA taken from the inset (e), (the color regions ranging from green to dark blue denote the compressive strain, while the regions from red to bright yellow represent the tensile strain).

We synthesized ordered‐phase multimetallic hexagonal nanoplates (NPLs) in nonaqueous conditions using Palladium(II) acetylacetonate [Pd(acac)_2_], Iridium (III) acetylacetonate [Ir(acac)_3_], Cobalt (II) acetylacetonate [Co(acac)_2_] and Copper (II) acetylacetonate [Cu(acac)_2_] as the metal precursors in formamide solution [details in the supplementary materials]. The structure and morphologies of the NPLs were characterized by powder x‐ray diffraction (XRD), transmission electron microscopy (TEM), high resolution TEM (HRTEM), aberration‐corrected high‐angle annular dark‐field scanning transmission electron microscopy (HAADF‐STEM) and STEM‐electron energy‐loss spectroscopy (EELS) techniques, which corroborates the alloy structure formation of NPLs under the given conditions. **Figure** **1** and Figure S1 (Supporting Information) show the TEM, HRTEM, HAADF‐STEM and mapping images of the PdCuIrCo NPLs. The HAADF‐STEM image taken from an individual NPL is shown in (Figure 1e), where the average spacings of two orthogonal lattice fringes are 0.29 and 0.21 nm, respectively, in good agreement with the experimental powder diffraction values of (100) and (110) d‐spacings for the ordered‐phase. The unique atomic arrangement (ABAB stacking) in the ordered structure of PdCuIrCo NPL can be readily observed from the HAADF‐STEM image (Figure 1e). The corresponding FFT pattern further affirms the ordered‐phase (Figure 1c). As revealed by the EELS mappings (Figure 1g) and line scan (Figure 1h), all the elements (Pd = Cyan, Cu = Magenta, Ir = Orange, Co = Red) are distributed in the whole NPL evenly, confirming the formation of PdCuIrCo alloy. The XRD pattern confirms the formation of the ordered body‐centered‐cubic (bcc) phase (Figure 1f). The diffraction pattern coincides well with the standard reference pattern of PdCu ordered‐phase (ICDD: 01‐078‐4406), Ir (JCPDS # 01–1212) and Co (JCPDS # 15–0806) phase. The appearance of characteristic peaks at 2θ = 29.4° and 42.5° confirms the ordered intermetallic structure of the PdCuIrCo NPLs.^[^ ^4^, ^6^, ^12^ ^]^ Next, we performed geometric‐phase analysis (GPA) to explore the distribution and extent of lattice strain in NPLs. The strain mappings of the selected area of the PdCuIrCo NPL are dominated mainly by the tensile‐strain (Figure 1i tensors and Figure S2, Supporting Information). The thickness of the PdCuIrCo NPLs was further investigated by atomic force microscopy (Figure S3, Supporting Information) and NPL has a thickness of ≈2 nm. Interestingly, by reducing one metal precursor (cobalt), while keeping other synthetic conditions same, we have obtained ordered‐phase PdCuIr NPLs (Figure S4, Supporting Information). TEM, HRTEM, HAADF‐STEM, EELS mapping and line scan images revealed the alloy formation. Moreover, XRD pattern confirms their ordered‐phase. The “ABAB” atomic stacking mode observed in the HAADF‐STEM image and the diffraction spots in the FFT pattern further confirm the formation of ordered‐phase (Figure S4, Supporting Information). From GPA, we found that the tensile strain also dominates the trimetallic nanoplate (Figure S5, Supporting Information).

The chemical structure of the PdCuIr and PdCuIrCo NPLs was characterized by X‐ray photoelectron spectroscopy (XPS) to enable investigating the surface composition, electronic structure, and interactions between elements (Figure S6, Supporting Information). The Pd 3d spectra of NPLs show two peaks that can be assigned to the Pd 3d_5/2_ and Pd3d_3/2_ and be further split into two doublets, associated with Pd^0^ and Pd^2+^ chemical states. The Cu 2p_3/2_ can be split into one doublet, associated with Cu^0^ and Cu^+^ chemical states. It is clear that the majority of the Pd and Cu atoms of the NPLs are mainly in the metallic state. Similarly, Ir 4f spectra also show two pairs of doublets, associated with Ir^0^ 4f_7/2_, Ir^0^ 4f_5/2_, Ir^4+^ 4f_7/2_, Ir^4+^4f_5/2_, respectively. Furthermore, the Co 2p spectra of PdCuIrCo NPLs show two different states. Noted that both Pd 3d spectra of PdCuIrCo NPLs are negatively shifted to higher binding energies compared with those of PdCuIr NPLs, while the intensity of Ir^4+^ 4f spectra of PdCuIrCo NPLs increased. Such peak shift visualizes a change in the surface electronic structure of tetrametallic NPLs, originated from charge transfer from Ir or Co to Pd or Cu. Furthermore, extended X‐ray absorption fine structure spectroscopy (EXAFS) was employed to investigate the chemical environments and electronic states of the elements of the ordered‐phase NPLs (Figure S7, Supporting Information). X‐ray absorption near‐edge structure (XANES) spectra at Pd K‐edge show that, the absorption threshold position of Pd were close to the metallic Pd(0) foil (Figure S7a, Supporting Information), and Pd‐M (Co, Cu, Ir) bonds can be found in WT transferred R space (Figure S7b, Supporting Information). While for Cu K‐edge, both Cu─O and Cu─M bonds can be found in WT transferred R space and the intensity of Cu─O in PdCuIrCo NPLs is more vital than that of PdCuIr NPLs (Figure S7c, Supporting Information). Furthermore, the XANES spectra shows that the absorption threshold position of Ir and the white line position of Ir for PdCuIrCo and PdCuIr were close to that of IrO_2_, implying the similar oxidation states. Similarly, Co for PdCuIrCo also shows Co─O bond (Figure S7h, Supporting Information). In addition, the atomic ratio of Pd/Cu/Ir/Co in PdCuIr and PdCuIrCo NPLs is 42.21:42.32:15.27 and 31.42:31.00:18.10:19.48 (Tables S1 and S2, Supporting Information), determined by inductively coupled plasma mass spectrometry (ICP‐MS). Thus, HRTEM, XRD, XPS and EXAFS results proved that ordered‐phase multimetallic alloy NPLs could be obtained successfully via a wet‐chemical method.

**Figure 2:**
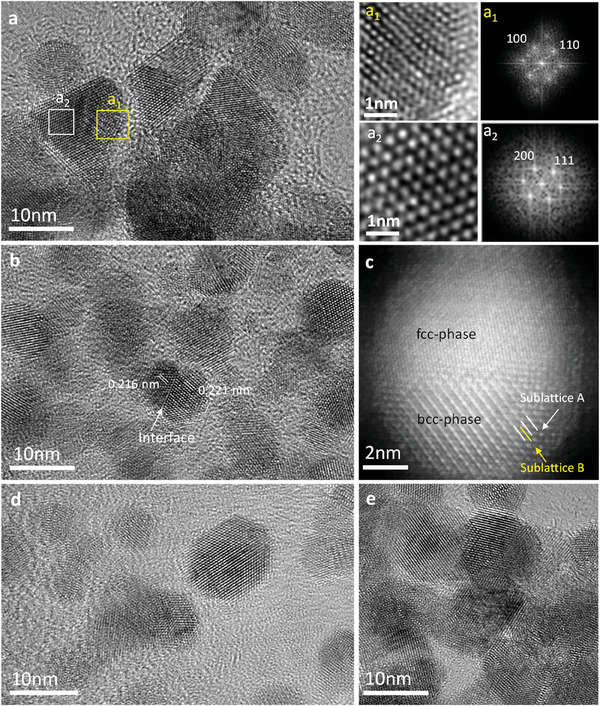
HRTEM images of PdCuIr nanocrystals obtained at different reaction time: a) at 5 min, a_1_, a_2_) HRTEM images with the corresponding FFT pattern taken from the dashed squares in (a), b,c) at10 min with its HAADF‐STEM image, d) at 20 min and e) at 30 min.

To obtain a deeper insight into the formation of the tri‐ and tetrametallic bcc phase NPLs, a series of control experiments were conducted. It was found that the products were PdCu and PdCuCo nanoparticles (Figures S8 and S9, Supporting Information) when no Ir was added in the reaction system. The XRD patterns corroborate the alloy structure and indicate the face‐centered‐cubic (fcc) nature of the as‐prepared alloy nanoparticles. Similarly, the products were fcc PdIr and PdIrCo alloy NPLs when no Cu was added (Figures S10 and S11, Supporting Information). While, without Pd precursor, no discernible morphology was observed (Figure S12, Supporting Information). As a result, along Pd the presence of Cu and Ir is the key factor in forming ordered‐phase PdCuIr and PdCuIrCo NPLs. The formation of tri‐ and tetrametallic ordered‐phase could be due to a synergistic effect of Pd, Cu, and Ir, and a detailed mechanism needs to be further explored. Therefore, we characterized the intermediate NCs of NPLs at different reaction durations by HRTEM and ICP‐MS. It should be pointed out that, capturing the structures of seeds involved in synthesizing PdCuIr and PdCuIrCo NCs is almost impossible, as their formation occurs at ultrahigh speeds.^[^ ^10^ ^]^ Moreover, the ordered‐phase can be clearly observed in the products obtained at 30 and 60 min reaction time (Figures S13–S15, Supporting Information). Notably, at 30 min reaction time the concentration of Co in PdCuIrCo NCs was lower compared to Pd, Cu, Ir and could be attributed to the slow reduction kinetics of Co.^[^ ^13^, ^14^ ^]^ Therefore, we performed experiments of PdCuIr NCs only, collected below 30 min reaction durations better to understand the formation process of order phase within NPLs. HRTEM images and FFT patterns of the NCs in **Figure** **2** show how the ordered‐phase evolves. It turns out that after 5 min reaction time, the shape of PdCuIr NCs was triangular NPLs and ordered‐phase nucleates on the NPLs surface (Figure 2a yellow marked area). As the reaction time reached 10 min, the ordered‐phase propagates across the NPLs (Figure 2b). With the increasing reaction time (10–20 min), the ordered‐phase propagates by deforming the fcc‐phase (Figure 2d; Figure S16, Supporting Information). When the reaction time reached 30 min, the product became hexagonal NPLs that fully transformed into an ordered‐phase (Figure 2e; Figure S14, Supporting Information).

Note that in the images series viewed from 10–20 min reaction time, the two phases are separated by a well‐defined sharp interface (Figure 2c; Figure S17, Supporting Information). The crystalline features of the fcc‐ phase and bcc‐phase appear differently (Figure 2b). The lattice spacing of 0.221 nm can be assigned to the (110) planes of bcc‐phase and the lattice spacing of 0.216 nm can be assigned to the (111) planes of fcc‐phase. However, the interface disappeared when the NPLs were viewed at 30 min (Figure 2e). At this stage, interestingly, the Ir content of the product increased to 13 wt% (Table S1, Supporting Information).

**Figure 3:**
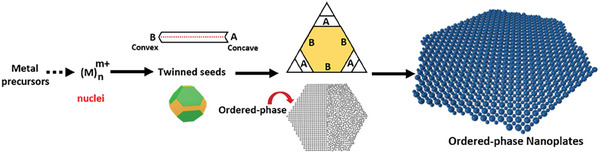
Schematic illustration of the ordered‐phase nanoplates.

It is clear that the Ir content may play an important role for the formation of ordered‐phase hexagonal NPLs (Figure S18, Supporting Information). The interface between the fcc and bcc phases is coherent interface, meaning the lattice planes of the two phases are continuous across the interface. The significance of the coherent interfaces is that the two phases appear due to the formation of twin nanoparticles at the early stage of the reaction when they are generated at high speeds.^[^ ^10^ ^]^ More specifically, the observation that ordered‐phase NPLs can form indicates that the formation of twinned nanocrystals controls the phase.^[^ ^15^ ^]^ These results and ICP‐MS analysis have led us to hypothesize that, important crystallographic considerations derive the growth of ordered phases in our reaction system. Among the precursors of our reaction system, Pd alloy can easily form twinned crystal seeds.^[^ ^16^, ^17^ ^]^ It should be pointed out that, there is no direct evidence to prove the structures of seeds involved in synthesizing PdCuIr and PdCuIrCo nanostructures. Our understanding of the structures of the seeds is mainly based on what we learned from the Pt and Ag systems. We believe this analogy is reasonable, because the reaction system and morphologies of the final products are more or less identical to our reported works.^[^ ^14^, ^18^, ^19^, ^20^ ^]^ As proposed by Lofton and Sigmund,^[^ ^21^ ^]^ the presence of a defect can cause the six side faces. Therefore, the twin defect leads to concave‐ and convex‐type faces in the hexagonal nucleus, and the preferential growth at the concave face (reentrant grooves) eventually leads to a triangular plate.^[^ ^18^, ^22^ ^]^ These reentrant grooves serve as a primary site for atomic addition, facilitating the transformation of triangular NPLs into ordered‐phase hexagonal NPLs.^[^ ^18^ ^]^ Moreover, coherent potential approximation calculations suggest that, at equal atomic percentages of Pd and Cu, the formation of an ordered‐phase in the alloy is energetically favored.^[^ ^4^, ^23^ ^]^ Our results indicate that (Figure S19, Supporting Information), the addition of Ir leads to equal atomic percentages of Pd and Cu and changes the structure of the alloy into ordered‐phase NPLs. From the aforementioned discussion it seems that incorporation of Ir could significantly alter the reduction kinetics and crystallinity of seeds, inducing the formation of novel ordered‐phase PdCuIr and PdCuIrCo nanoplates. In addition, it has also been found that sizes and surface properties of nuclei may determine their growth behavior.^[^ ^18^, ^19^ ^]^ Here the gel‐like material may act as a template for the formation of smaller nuclei, which then lead to the growth modes of 2D alloy nanoplates (Figures S20 and S21, Supporting Information).^[^ ^14^ ^]^ Thus, results demonstrate that the ordered‐phase nucleates through crystallinity alteration of the seeds and then propagates by reentrant grooves and eventually forming ordered‐phase NPLs. The proposed growth mechanism is schematized in **Figure** **3**.

**Figure 4:**
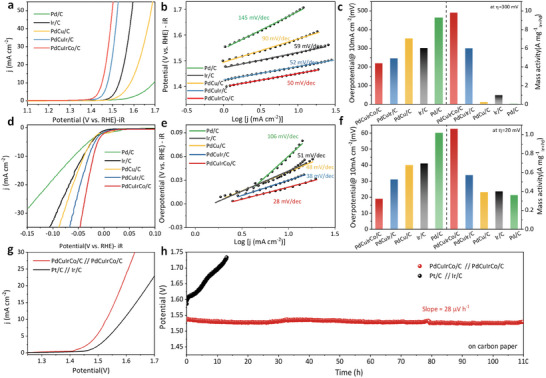
The electrocatalytic water splitting performance of different electrocatalysts in O_2_ saturation 0.1 m HClO_4_. a) The OER polarization curves recorded with a linear scan of potential at 5 mV s^−1^ from the PdCuIrCo/C, PdCuIr/C, PdCu/C, Pd/C, and Ir/C respectively. b) Tafel plot of different electrocatalysts. c) The overpotential at a current density of 10 mAcm^−2^ (left) and mass activity at an overpotential of 300 mV (vs RHE) of different electrocatalysts (right). d) The HER polarization curves recorded with a linear scan of potential at 5 mV s^−1^ from the PdCuIrCo/C, PdCuIr/C, PdCu/C, Pd/C, and Ir/C, respectively. e) Tafel plot of different electrocatalysts. f) The overpotential at a current density of 10 mAcm^−2^ (left) and mass activity at an overpotential of 20 mV (vs RHE) of different electrocatalysts (right). g) The LSV of PdCuIrCo/C // PdCuIrCo/C and Pt/C // Ir/C. h) The galvanostatic curves of PdCuIrCo/C // PdCuIrCo/C and Pt/C // Ir/C at a current density of 10 mA cm^−2^.

As known, ordered intermetallic alloys have attracted extensive attention as advanced electrocatalysts for fuel cell reactions with much improved activity and stability. As a proof‐of‐concept application, the as‐synthesized ordered‐phase PdCuIr and PdCuIrCo NPLs were tested for the OER and HER using a conventional three‐electrode setup to demonstrate their structural advantage in an acidic electrolyte (0.1 m HClO_4_). To evaluate the catalytic performance, the as prepared ordered‐phase nanoplates were loaded onto a carbon black support (Vulcan XC‐72) and denoted as PdCuIr/C and PdCuIrCo/C respectively. For comparison, PdCu/C nanoparticles, commercial Pd/C and Ir/C catalysts were also assessed. The OER and HER activities of the PdCu/C, PdCuIr/C, and PdCuIrCo/C catalysts were compared with that of commercial Pd/C and Ir/C (20 wt%). The noble metals (Pd and Ir) contents in the as‐prepared catalysts were determined by ICP‐MS analysis (Table S3, Supporting Information), and their loading on the electrode was controlled at 10.2 ug cm^−2^. We used cyclic voltammetry (CV) to measure the electrochemically active surface area (ECSA) of each electrocatalyst (Figure S22a, Supporting Information), which are in the following order: Pd/C > PdCu/C > PdCuIrCo/C > PdCuIr/ C > Ir/C. The OER performance of as‐prepared catalysts together with that of the commercial Ir/C and Pd/C was conducted in an O_2_‐saturated electrolyte at a scan rate of 5 mV s^−1^ (**Figure** **4**a). The OER polarization curves of the catalysts clearly revealed enhanced OER activities of ordered‐phase catalysts as compared to commercial catalysts (Table S4, Supporting Information). It can be observed that, the required overpotential of PdCuIrCo/C and PdCuIr/C is 221 and 247 mV to achieve a current density of 10 mA cm^−2^, significantly lower than that of Ir/C (301 mV), PdCu/C (354 mV), and Pd/C (465 mV) respectively. This suggests that the ordered structure of the as‐prepared catalysts plays a significant role in the OER process.^[^ ^8^, ^9^, ^15^ ^]^ Remarkably, the measured overpotential of PdCuIrCo/C (221 mV) is superior to the other recently reported noble‐metal based electrocatalysts in acidic media (Table S5, Supporting Information). Further analysis of the corresponding Tafel slopes was conducted and PdCuIrCo/C exhibited the lowest Tafel slope value (50 mV dec^−1^) (Figure 4b). As shown in Figure 1c, the PdCuIrCo/C exhibited a mass activity of 9.8 A mg^−1^ at the overpotential of 300 mV, which was 1.6, 10.0, 37.7, and 140 times higher than those of PdCuIr/C (6.00 A mg^−1^), commercial Ir/C (0.98 A mg^−1^), PdCu/C (0.26 A mg^−1^) and commercial Pd/C (0.07 A mg^−1^), respectively. This confirms the substantial improvement of the OER activity of the ordered‐phase structure. We then turned our attention to the HER performance of the ordered‐phase NPLs and found that the unique PdCuIr/C and PdCuIrCo/C catalysts also deliver excellent HER performance (Figure 4d). The HER performance of as‐prepared catalysts and the commercial Ir/C and Pd/C was conducted in N_2_‐saturated electrolyte at a scan rate of 5 mV s^−1^. As shown by the linear sweep voltammetry (LSV) curves (Figure 4d), the ordered‐phase PdCuIrCo/C and PdCuIr/C catalysts exhibited remarkable superiorities compared to the commercial Pd/C and Ir/C catalysts (Table S6, Supporting Information).

The overpotential at the current density of 10 mA cm^−2^ of PdCuIrCo/C was 19 mV, much lower than that of PdCuIr/C (31 mV), PdCu (40 mV), Ir/C (41 mV), and Pd/C (60 mV). This demonstrates ordered‐phase NPLs as an outstanding HER electrocatalyst. Further analysis of the corresponding Tafel slopes was conducted to gain a better understanding of the HER catalytic reaction. The Tafel slope of PdCuIrCo/C is 28 mV dec^−1^, much lower than that of PdCuIr/ C (38 mV dec^−1^), PdCu/C (48 mV dec^−1^), Ir/C (51 mV dec^−1^), and Pd/C (60 mV dec^−1^) (Figure 4e). Furthermore, the mass activity at the overpotential of 20 mV of PdCuIrCo/C was 1.06 A mg^−1^, which still exhibited the highest mass activity among the four samples (Figure 4f). In addition, the PdCuIrCo/C is also superior to commercial Pt/C (Figure S23, Supporting Information) and recently reported noble‐metal based electrocatalysts (Table S7, Supporting Information) representing the enhanced HER activity of ordered‐phase catalyst in acidic electrolyte. We also compared the OER and HER activities of the PdCuIrCo/C at different loading amount onto the electrode. It was found that by increasing catalyst loading on RDE from 5 to 20 ug cm^−2^, the overpotential can be reduced (Figure S24, Supporting Information). As described above, the ordered‐phase PdCuIrCo/C catalyst exhibits significant improvements either in OER or HER compared with PdCu/C, Pd/C, and Ir/C catalysts, we subsequently measured the overall water electrolysis using PdCuIrCo/C as both the cathode and anode catalysts in a standard two‐electrode system. As shown by the polarization curves (Figure 4g), the PdCuIrCo/C exhibits a potential of 1.53 V to acquire 10 mA·cm^−2^ current density in 0.1 m HClO_4_, which is much lower than that of the benchmark Pt/C||Ir/C electrode couple (1.59 V). We further investigated the long‐term stability of the PdCuIrCo/C by chronoamperometry (CP) technique. At a current of 10 mA cm^−2^, the system continues to operate for >110 h with a potential increase of only 28 µV h^−1^ (Figure 4h), further revealing that the bifunctional PdCuIrCo/C catalyst possesses outstanding long‐term durability for overall water splitting. This better stability of catalyst could be due to the stable morphology, surface structure and low diffusion rates and high bond energies of the ordered‐phase NPLs during the reaction (Figure S25, Supporting Information).^[^ ^24^ ^]^ This indicates that the ordered‐phase of the catalyst could hinder the dissolution of the elements, which was beneficial in improving the stability of the catalyst.^[^ ^2^ ^]^ In addition, during the stability test, the massive evolution of H_2_ and O_2_ on both the cathode and anode (Figure S26, Supporting Information), intuitively demonstrated the excellent overall water splitting performance of ordered‐phase catalyst. These results indicate that the ordered‐phase of NPLs can intensify structural and catalytic stabilities.^[^ ^1^, ^11^ ^]^ Upon identifying the improved overall water splitting performance of ordered‐phase NPLs, we have pointed out several reasons behind this process.

The high activity of ordered‐phase NPLs (PdCuIr and PdCuIrCo) is attributed to their unique structure, alloy composition and ordered‐phase hexagonal morphology.^[^ ^11^, ^25^, ^26^, ^27^ ^]^ The hexagonal morphology endows the particles with atomic ordering at surfaces, which can expose more active sites and bring high utilization of the Pd and Ir atoms. Furthermore, the introduction of Co can affect the electronic structure of NPLs by the so‐called electronic and ligand effects by alloying with other elements, thus tuning the binding energy to the intermediates.^[^ ^14^, ^28^ ^]^ Nevertheless, the Ir ratio is increased after Co species are introduced (Table S2, Supporting Information), suggesting an increased number of active sites and more charge polarization on Ir in PdCuIrCo NPLs than in the case of the trimetallic PdCuIr NPLs, thus promoting the electrocatalytic performances of NPLs which is in agreement with already reported work.^[^ ^29^ ^]^ Further to examine the characteristic of the ordered‐structure, we compared the OER and HER activities of the PdIr and PdIrCo nanocrystals (Figure S27, Supporting Information). The results reflected that the ordered‐structure of the PdCuIrCo NPLs is beneficial to enhance the activity. In addition, the electrochemical performance of PdCuIrCo NPLs without carbon support was investigated (Figure S28, Supporting Information). Obviously, the overpotential at the current density of 10 mA cm^−2^ of PdCuIrCo/C was much lower than that of PdCuIrCo without carbon support.

**Figure 5:**
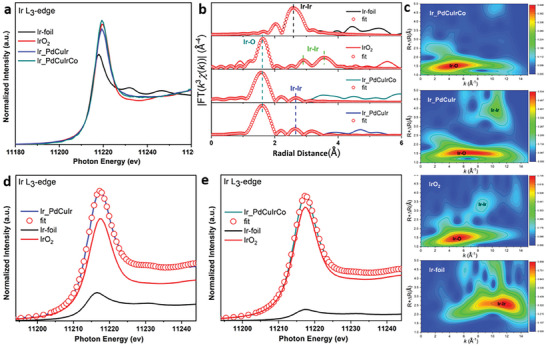
Electronic structural characterization of nanoplates. a) Experimental XANES spectra at the Ir L_3_‐edge. b) Ir L_3_‐edge Fourier transformed (FT) k^3^‐weighted χ(k)‐function of the EXAFS spectra. c) The wavelet transformed spectra of as‐prepared catalysts, d,e) Ir L_3_‐edge XANES Linear combination fitting of PdCuIrCo and PdCuIr.

Since the Ir in the ordered‐phase promotes the electrocatalytic performances of NPLs, the EXAFS was carried out on PdCuIr and PdCuIrCo NPLs to learn more about the active sites of catalysts (**Figure** **5**). As for the Ir L_3_‐edge XANES spectra, Figure 5a show that the intensity of white lines of PdCuIrCo and PdCuIr NPLs are much higher than Ir foil but close to IrO_2_ that indicated that the Ir in both catalysts are more inclined to exist in oxidative states. Compared with PdCuIr NPLs, the intensity of the white line of PdCuIrCo NPLs is higher which means that Ir has a higher coordinative number, due to the incorporation of Co.^[^ ^29^ ^]^ The corresponding fourier transform of the Ir L_3_‐edge EXAFS of PdCuIrCo NPLs also showed that Ir‐O bond was found clearly. Almost no Ir–Ir (≈2.5 Å) was observed which further confirmed the higher oxidative state of Ir in PdCuIrCo NPLs. These results corroborate that highly dispersed Ir^x+^ species exist as IrO_2_ resemblance.^[^ ^25^, ^29^, ^30^, ^31^ ^]^

In addition, the fitting results showed that the coordination number (CN) of O atoms in the first shell of Ir for the PdCuIr and PdCuIrCo NPLs were estimated to be 4.88 and 5.4 at a distance of 2.00 Å respectively (Table S8, Supporting Information), which are lower than that of the standard IrO_2_ (CN = 5.9), indicating that unsaturated Ir atoms exposed on the surface of catalysts to achieve high catalytic performance, which is consistent with earlier reported Ir‐based works.^[^ ^30^, ^31^, ^32^ ^]^ Concurrently, the bond distance is slightly stretched (Table S8, Supporting Information), and induces tensile‐strain.^[^ ^3^, ^30^, ^32^ ^]^ To more precisely identify the oxidation states for the Ir species, wavelet transform (WT) analysis was also carried out (Figure 5c). The WT of EXAFS for Ir in Figure 5c presents a negative shift toward Ir–O suggesting that Ir of PdCuIrCo NPLs is more inclined to oxidized states (IrO_x_). To further quantitative analyze the oxidation state distribution of Ir in ordered‐phase catalysts, we conducted linear combination fitting for Ir L_3_‐edge XANES (Figure 5d,e). The results showed that, for Ir *L_3_*‐edge of PdCuIrCo NPLs, 95.3% can be attributed to the oxidative state Ir (+4). Only 4.7% can be attributed to Ir (0) (Table S9, Supporting Information). Regarding PdCuIr NPLs, the contribution of Ir (0) is 21.6%, about five times as much as that of PdCuIrCo NPLs. These fitting results confirmed that adding of Co induces the formation of a higher oxidation state Ir in PdCuIrCo NPLs. This suggests that the Ir species with a high valence state act as the active sites of NPLs for OER and HER application.^[^ ^31^, ^32^, ^33^, ^34^ ^]^ Another reason for the better catalytic performance of the ordered‐phase NPLs can be primarily attributed to the shear strain effects that contribute to the optimal adsorption of the reaction intermediates.^[^ ^20^, ^35^ ^]^ It is found that, the ordered atomic arrangement in the intermetallic structures of the multimetallic NPLs endows intensified strain leading to the enhanced overall water splitting performance.^[^ ^30^, ^36^, ^37^ ^]^ As shown in Figure S29 (Supporting Information), the tensile‐strain arising from the expansion of the lattice length in the x and y directions on the surface of the ordered‐phase NPLs improves catalysis.^[^ ^30^ ^]^ This result agrees with the XAS analysis of nanoplates.

**Figure 6:**
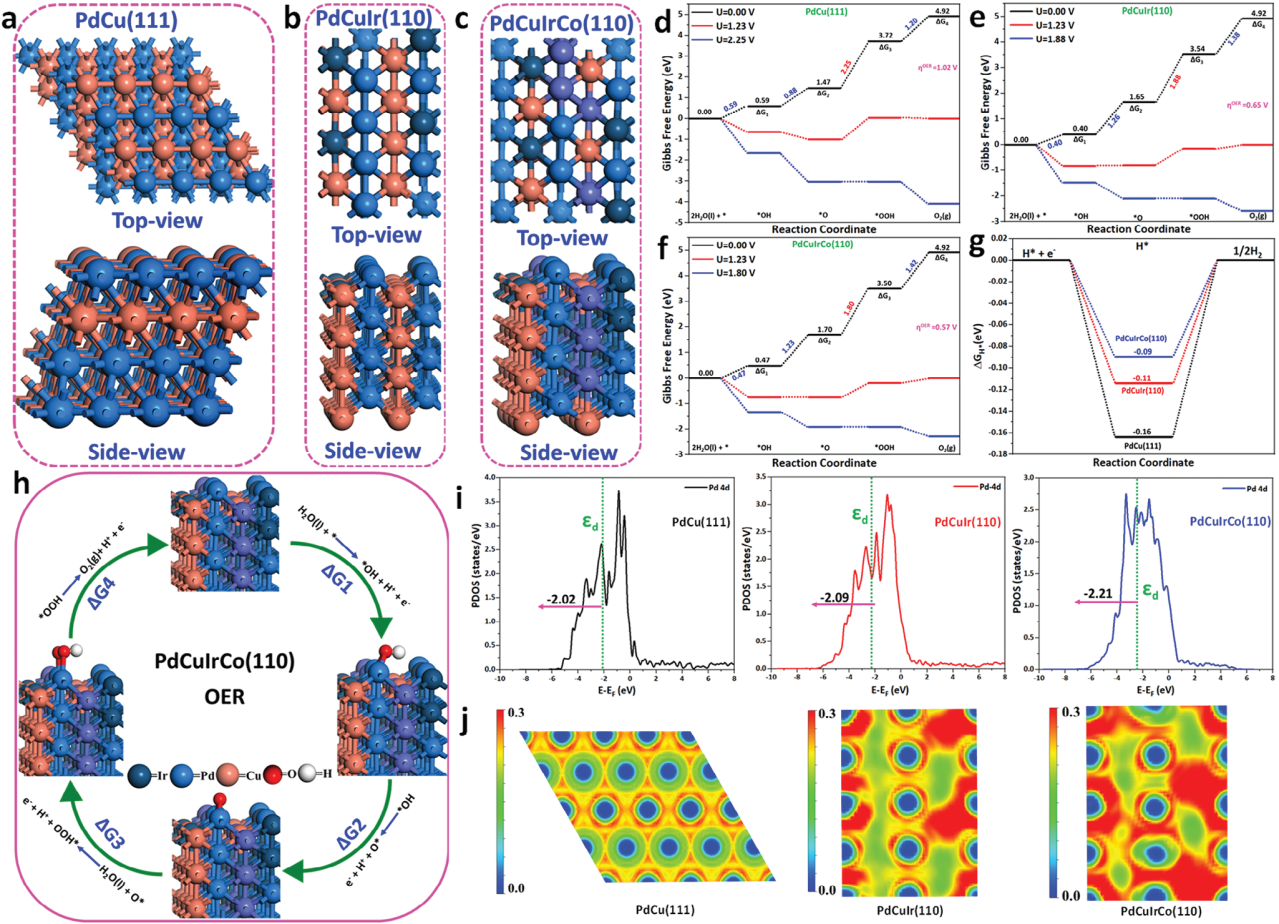
a–c) Top and side views of the optimized configuration of PdCu (111), PdCuIr (110) and PdCuIrCo (110) NCs. d–f) Calculated the potential free energy profile of the intermediate states for OER on the PdCu (111), PdCuIr (110) and PdCuIrCo (110) NCs. g) Gibbs free energy profile (∆G_H*_) for HER at active site on the NCs. h) Schematic of the 4e^−^ OER mechanism of the active site on the NCs with the optimized configurations for intermediates, the absolute value of ∆G_H*_ for HER activity is close to zero (∆G_H*_ → 0). i) Calculated spin‐polarized partial density of states (PDOS) of the d‐band for the surface Pd‐atoms in the PdCu (111), PdCuIr (110) and PdCuIrCo (110) NCs. The green dashed lines specify the calculated d‐band center. j) The calculated electron localization function (ELF) of PdCu (111), PdCuIr (110) and PdCuIrCo (110) NCs.

To further elucidate the role of ordered‐phase structure in promoting OER and HER performance of NPLs, we applied density functional theory (DFT) calculations. Our study of the OER activity of stable PdCu (111) nanocrystals (NCs) before and after doping Ir and co‐doping IrCo atoms provided insight into the excellent OER performance of the PdCuIrCo (110) surface. We used a computational standard hydrogen electrode (SHE) model combined with self‐consistent theoretical overpotential methods to analyze the OER activity. To investigate the mechanism of OER, we first constructed surface Pourbaix diagrams of PdCu (111), PdCuIr (110), and PdCuIrCo (110), which are comprised of the most stable surface phases at the electrode potentials, considering the harsh conditions of OER (**Figure** **6**a–c). In this scenario, a balance is achieved between PdCu (111), PdCuIr (110), and PdCuIrCo (110), as well as water, protons, and electrons through the following equations:

(1) $$H_{2}O{+}^{\;*\;}\to \;H^{+}+\;e^{-}{+}^{\;*}\mathrm{OH}$$

(2)

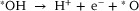




where * represents the active‐site of the catalyst. Based on the DFT results in Figure 6a–c, the exposed Pd‐atoms on these three NCs are chemically active toward H_2_O under OER working conditions, leading to easy surface oxidation. A potential‐limiting step is determined by calculating the Gibbs free energies (ΔG) of OH*, O*, and OOH* species on PdCu (111) NCs, PdCuIr (110), and PdCuIrCo (110) NPLs. Figure 6d–f, presents a self‐consistent reaction scenario, indicating that OER can proceed on the specified surface under a stable potential. These intermediates were adsorbing on the nanoplate models (Figure 6h; Figures S30 and S31, Supporting Information), i.e., PdCu (111), PdCuIr (110), and PdCuIrCo (110). The appropriate overpotential (η^OER^) value can be derived from Equation S6 (Supporting Information) based on the four‐intensive adsorption/desorption processes and well‐defined active sites. As shown in Figure 6d–f, the calculated overpotential on PdCu (111) NCs, PdCuIr (110), and PdCuIrCo (110) NPLs were 1.02, 0.65, and 0.57 V, respectively, with the formation of *O_2_ → *OOH being the potential‐limiting step (PDS). This phenomenon may be due to the strong bond between *O and Pd‐atoms. In addition, the PdCuIrCo (110) NPLs shows the lowest overpotential (0.57 V) compared with the PdCu (111) NCs and PdCuIr (110) NPLs, indicating efficient OER activity. However, IrCo co‐doping in PdCu (111) NC significantly adjusts the binding of the OER intermediates on PdCuIrCo (110), as evidenced by the weakened adsorption energies of *OH, *O, and *OOH, compared with those on PdCu (111) and PdCuIr (110). This result suggests that IrCo co‐doped in PdCu (111) NC can provide efficient catalytic performance for OER. The above‐mentioned results indicate that the ordered‐phase PdCuIrCo (110) is a promising OER model with good agreement with experimental data.

For HER performance on PdCu (111) NCs, and PdCuIr (110), PdCuIrCo (110) NPLs, DFT‐based modeling under standard conditions was also performed to understand the nature of the reaction mechanism and active site at the atomic level. The Gibbs Free energies for the adsorption of atomic hydrogen (ΔG_H*_) on these NCs at active sites have been investigated as presented in Figure 6g. The ΔG_H*_ of an ideal HER system should be near zero (ΔG_H*_ → 0). The PdCuIrCo (110) NPLs shows near zero ΔG_H*_ (−0.09 eV), a little smaller than that for PdCuIr (110) NPLs (−0.11 eV) but lower in magnitude than the PdCu (111) NCs (−0.16 eV), which is consistent with the experimental finding in Figure 4. Furthermore, the PDOS of the d‐band for Pd‐atoms on the NCs of PdCu (111), and PdCuIr (110), PdCuIrCo (110) NPLs were compared (Figure 6i). For the Ir‐doped and IrCo co‐doped compounds, the d‐band center of PdCuIr (110) and PdCuIrCo (110) NPLs downshifted by 0.07 and 0.19 eV relative to that on PdCu (111) NCs, respectively. It is found that, the d‐band focus of Pd on the PdCuIrCo (110) NPLs downshifts by 0.19 eV, indicating that the PdCuIrCo (110) NPLs has the higher activity for OER and HER. The relation between the d‐band center of the Pd‐atom and overpotential of OER and HER is shown in Figure S32 (Supporting Information). It was observed that the d‐band center of Pd‐atom shifted far away from the Fermi level (E_F_), which clearly shows the weak interaction between selected catalysts and key intermediates (*OH, *O, *OOH, and H*) for OER and HER. This validates that the Pd‐atom significantly promotes the HER and OER activity of PdCuIrCo (110) NPLs. The electron localization function (ELF) of PdCu (111) NCs and PdCuIr (110), PdCuIrCo (110) NPLs were calculated to further understand the enhanced catalytic activity of the Pd site adjacent to the Cu atom (Figure 6j). It is important to note that the ELF values are inversely proportional to their covalent and electrostatic interactions. The ELF plot can be used to visualize the probability of finding electrons near a reference atom. Depending on the ELF value, a high value (green region) means there is a high likelihood of finding electron localization, and a red region implies electron gas. Figure 6c shows how the electronic configuration of surface atoms changes and reduces after co‐doping CoIr in PdCu (111). Therefore, we conclude that the surface electronic locality follows the order of PdCuIrCo (110) > PdCuIr (110) > PdCu (111). Moreover, the localization of the excess electron density around the Pd site is the active site of NPLs. In general, ordered‐phase NPLs have more homogeneous active sites, which are beneficial to regulating OER and HER.

## 3 Conclusion

In summary, we have successfully synthesized ordered‐phase nanoplates (NPLs) by a facile wet‐chemical method. The key to the formation of intermetallic NPLs is the utilization of crystallinity of seeds and intermediate nanocrystals (reentrant grooves) that lead to the formation of ordered intermetallic PdCuIr and PdCuIrCo NPLs. Geometric‐phase analysis demonstrates that the ordered‐phase can induce tensile‐strain in NPLs, and X‐ray absorption spectroscopy discloses that the oxidized states of Ir in ordered‐phase NPLs have a significant impact on the electronic structure. Benefiting from the ordered‐phase with tensile‐stain, the multimetallic NPLs exhibits higher intrinsic activity toward the OER and HER. In particular, we have demonstrated by both theoretical calculations and experimental results that, ordered‐phase of NPLs may play a critical role in promoting the water electrolysis. As a result, ordered PdCuIrCo NPLs have improved OER and HER activity and exhibited significantly higher mass activity and durability (110 h) than those of commercial catalysts in acidic conditions. Thus, it is possible to synthesized multimetallic ordered‐phase NPLs and maximize their exposure of active‐sites on the surface that exhibit better OER and HER catalytic properties. We believe that the ordered‐phase NPLs can not only serve as a high‐efficiency catalyst for water electrolysis but also act as a model to explore further properties of ordered alloy materials.

## Conflict of Interest

The authors declare no conflict of interest.

## Supporting information

Supporting Information.

## Data Availability

The data that support the findings of this study are available from the corresponding author upon reasonable request.
