# Supplementary material for: Unveiling the Growth Mechanism of Ordered‐Phase within Multimetallic Nanoplates
Source: Adv Sci (Weinh). 2024 Feb 29;11(17):2309163. doi: 10.1002/advs.202309163 (PMC11077676; doi:10.1002/advs.202309163)
Supplement: Supplementary file 1 — Supporting Information. [file ADVS-11-2309163-s001.pdf]

ADVANCED SCIENCE
Open Access

Supporting Information

for *Adv. Sci.*, DOI 10.1002/advs.202309163

Unveiling the Growth Mechanism of Ordered-Phase within Multimetallic Nanoplates

Azhar Mahmood, Dequan He, Chuhao Liu, Shamraiz Hussain Talib, Bolin Zhao, Tianren Liu, Ying He, Lijuan Chen, Dongxue Han* and Li Niu**

Unveiling the Growth Mechanism of Ordered-phase within Multimetallic Nanoplates

Azhar Mahmood,*[,1,2] Dequan He,[1] Chuhao Liu,[4] Shamraiz Hussain Talib,[2,5] Bolin Zhao,[1] Tianren Liu, [1] Ying He,[1] Lijuan Chen,[1] Dongxue Han*[1] and Li Niu*[,1,3]

[1]Center for Advanced Analytical Science, Guangzhou Key Laboratory of Sensing Materials and Devices, Guangdong Engineering Technology Research Center for Photoelectric Sensing Materials and Devices, School of Chemistry and Chemical Engineering, Guangzhou University, Guangzhou 510006, P. R. China.

[2]Department of Chemistry, Tsinghua University, Beijing 100084, China

[3]School of Chemical Engineering and Technology, Sun Yat-sen University, Zhuhai 519082

[4]College of Chemistry and Molecular Engineering, Peking University, Beijing 100871, China

[5]Advanced Materials Chemistry Centre, Khalifa University of Science and Technology, Abu Dhabi, 127788, United Arab Emirates

Author Contributions

Azhar Mahmood, Dequan He and Chuhao Liu contributed equally.

Emails: azhar@gzhu.edu.cn; dxhan@gzhu.edu.cn lniu@gzhu.edu.cn; niuli@mail.sysu.edu.cn

Experimental Section:

Reagents: Palladium(II) acetylacetonate [$Pd(acac)_2$], Iridium (III) acetylacetonate [$Ir(acac)_3$], Cobalt (II) acetylacetonate [$Co(acac)_2$] and Copper (II) acetylacetonate [$Cu(acac)_2$] (PVP, MW = 10,000), tris(hydroxymethyl) aminomethane (99.8%), formaldehyde (HCHO) solution (37%) and formamide (99.5%) were purchased from Sigma-Aldrich. Acetone (99.9%) and ethanol (99.9%) were purchased from Sinopharm Chemical Reagent. All the materials were used as received without further purification for the Synthesis of multimetallic nanocrystals.

Characterization: The X-ray diffraction (XRD) were performed using a PW3040/60 PANalytical with Cu Kα as an X-ray source. The transmission electron microscopy (TEM), High-angle annular dark-field (HAADF) STEM images and the corresponding energy dispersive spectrometer (EDS) were collected on a JEOL JEM-2100F 200kV field emission transmission electron microscope equipped with an OXFORD EDS. The X-ray photoelectron spectroscopy (XPS) were collected on ESCALAB 250Xi with Al Kα radiation (1486 eV) as a probe. The content of Pd,Cu,Ir and Co were determined by the ICP-AES (PerkinElmer NexION 300X). The X-ray Absorption Near Edge Structure (XANES) and Extended X-ray Absorption Fine Structure (EXAFS) data were collected at Beamline 11B at Shanghai Synchrotron Radiation Facility (SSRF).

Synthesis:

Synthesis of PdCuIrCo Nanoplates: In a typical synthesis of PdCuIrCo nanoplates, a mixture of 100 mg of tris (hydroxymethyl) aminomethane and 400 mg of PVP was dissolved in 3 mL of HCHO solution and transferred to Teflon-lined stainless steel autoclave which was heated at 200 °C for 3 h. A gel-like material was obtained, after the washing and centrifugation in acetone. A homogeneous solution of 0.04 mmol of $Pd(acac)_2$, 0.04 mmol $Ir(acac)_3$, 0.04 mmol $Co(acac)_2$ and 0.04 mmol $Cu(acac)_2$ and 100 mg of KI was prepared in 10mL of formamide solvent and poured into a 25 mL Teflon-lined stainless steel autoclave along with the gel-ike material which was prepared before; the autoclave was then kept in the oven at 150 °C for 3 h. The final product was obtained after washing with ethanol and acetone.

Synthesis of PdCuIr Nanoplates: The synthesis procedure and experimental conditions for PdCuIr nanoplates were same as the PdCuIrCo nanoplates, except that, $Co(acac)_2$ precursor was not added the reaction solution.

Note: It is important to note that, for the explanation of formation mechanism of the ordered-phase nanoplates, we vary the reaction time (5-10 min, 20 min, 30 min and 3h).

Synthesis of PdCu, PdCuCo, PdIr, PdIrCo, IrCu and PdCuIrCo Nanostructures: The synthesis procedure and experimental conditions for PdCu, PdCuCo, PdIr, PdIrCo and IrCu nanostructures were same as the PdCuIrCo nanoplates. Note, PdCuIrCo nanocrystals were synthesized without gel-like materials.

Electrochemical Measurements:

The preparation of catalysts for electrochemical characterizations:

The as-prepared samples were loaded on carbon (Vulcan XC-72) by sonicating in cyclohexane for 2 h (20 wt% of the precious metal is loaded on Vulcan XC-72). 2 mg of as prepared PdCuIr/C, PdCuIrCo/C, and PdCu/C catalysts were then dispersed in a mixed solvent containing isopropanol, ultrapure water, and Nafion (the volume ratio is 6/4/0.02) by sonication for 30 min to obtain a homogeneous ink with a concentration of 1 mg mL^{-1}.

Electrochemical characterization: All the electrochemical measurements were performed using an AUTOLAB PGSTAT302N potentiostat (Metrohm AG). A Pt wire and a reversible hydrogen electrode were applied as the counter and reference electrodes, respectively. The working electrode was prepared by dropping 10 μL of the catalyst ink onto a glassy-carbon electrode (GCE, 5 mm, 0.196 cm^2). The cyclic voltammetry (CV) curves of the catalysts were recorded at 30°C in an N_2-saturated 0.1 M $HClO_4$ solution in the potential range of 0.05−0.8 V at a scanning rate of 50 $mV\cdot s^{-1}$. The electrochemically active surface area (ECSA) of all catalysts were determined from the charges associated with desorption of hydrogen in the potential range of 0.05−0.4 V after double-layer correction. (cited by DOI: 10.1021/acs.chemmater.9b02011) The OER and HER polarization curves of the catalysts were collected at 30°Cin an O_2-saturated 0.1 M $HClO_4$ solution via the RDE method at an scanning rate of 5 $mV\cdot s^{-1}$ with a rotating speed of 2500 rpm with 95% Ohmic iR drop compensation. The chronopotentiometry curves were recorded at a constant current density of 10 mA cm^{-2} without IR-compensation. According to the former study, a certain amount of the catalyst ink was dropped onto the carbon fiber paper with a geometric area of 1 cm × 1 cm for preparing the electrodes for overall water splitting. The mass loading density of Ir in carbon paper was ~100 ug cm^{-2}. The mass loading density of Pt in carbon paper was ~50 ug cm^{-2}. The overall water splitting performance was collected in 0.1 M $HClO_4$ solution using the catalysts-modified carbon paper as both the anode and cathode in a two-electrode system.

Computational Methodology:

We used the Vienna ab initio simulation package (VASP 5.4.4) to execute all calculations based on spin-polarized density functional theory (DFT)[1-4]. A projector-augmented wave (PAW) method was used to describe electron-ion interactions. A generalized gradient approximation (GGA) with the Perdew-Burke Ernzerh (PBE) functional was employed to calculate electron exchange and correlation energy.[5] To avoid potential interactions between consecutive periodic images, the 15 Å vacuum space was used along the z-direction. A kinetic-cutoff energy of 400 eV was chosen for the plane wave basis. A convergence criterion of 1.0×10^{-6} eV/atom was set for the electronic energy, and ionic relaxation continued until the atomic force was less than 0.01 eV/Å. A semiempirical dispersion-corrected DFT+D3 scheme was used by Grimme to calculate the van der Waals interaction. The first Brillouin zone was sampled in the Monkhorst−Pack grid. The 3×3×1 *k*-point mesh was employed for geometric optimization and electronic structures analysis.[6] Based on the computational hydrogen electrode model, the Gibbs free energy change for each elementary step of HER and OER was calculated.

Steps for OER processes can be summarized as follows:[7]

$$M^* + O_2(g) + H^+ + e^- \rightarrow MOOH^* \qquad (S1)$$

$$MOOH^* + H^+ + e^- \rightarrow MO^* + H_2O(l) \qquad (S2)$$

$$MO^* + H^+ + e^- \rightarrow MOH^* \qquad (S3)$$

$$MOH^* + H^+ + e^- \rightarrow M^* + H_2O(l) \qquad (S4)$$

For the above elementary steps (ΔG_{OOH^*}, ΔG_{O^*}, ΔG_{OH^*}, ΔG_{H^*}) containing electron transfers, the Gibbs free energy difference would be calculated using the equations below:

$$\Delta G = \Delta E + \Delta E_{ZPE} - T\Delta S \qquad (S5)$$

where ΔE is the total energy change of intermediates adsorbed on the catalyst surface based on the DFT calculations, ΔZPE is the correction of zero-point energy and ΔS represents entropy contribution, respectively, and T is room temperature (298.15 K). The free energy of ($H^+ + e^-$) at standard conditions was assumed as the energy of $1/2H_2$.[8-9] The entropy of the H_2 is taken from the NIST[8] database, while the entropies of other intermediates were calculated from the vibrational frequencies.

Gibbs free energy (ΔG) for four elementary steps for OER processes is defined as follows: $\Delta G_a = \Delta G_{OH^*}$, $\Delta G_b = \Delta G_{OH^*} - \Delta G_{O^*}$, $\Delta G_c = \Delta G_{O^*} - \Delta G_{OOH^*}$, $\Delta G_4 = \Delta G_{OOH^*} - 4.92$.

Under electrode potential U = 0 V, the ΔG for all the four elementary steps can be calculated by:

i) $\Delta G_a = G(HO^*) + G(H^+ + e^-) – G(H_2O) – G(*)$

Or $\Delta G_a = G(HO^*) + 1/2G(H_2) – G(H_2O) – G(*)$

$= (E_{OH*} + ZPE_{OH*} - TS_{OH*}) +1/2(E_{H2} + ZPE_{H2} - TS_{H2}) - (E_{H2O} + ZPE_{H2O} - TS_{H2O}) – E^*$

ii) $\Delta G_b = G(O^*) + G(H^+ + e^-) - G(OH*)$

Or $\Delta G_b = G(O^*) + G(H_2) - G(OH*)$

$= (E_{O*} + ZPE_{O*} - TS_{O*}) +1/2(E_{H2} + ZPE_{H2} - TS_{H2}) - (E_{OH*} + ZPE_{OH*} - TS_{OH*})$

iii) $\Delta G_c = G(HOO^*) + G(H^+ + e^-) – G(H_2O) - G(O*)$

Or $\Delta G_c = G(HOO^*) + 1/2G(H_2) – G(H_2O) - G(O*)$

$= (E_{OOH*} + ZPE_{OOH*} - TS_{OOH*}) +1/2(E_{H2} + ZPE_{H2} - TS_{H2}) - (E_{O*} + ZPE_{O*} - TS_{O*})$

iv) $\Delta G_d = G(O_2) + G(H^+ + e^-) – G(OOH*)$

Or $\Delta G_d = \{4.92 + 2G(H_2O) -2G(H_2)\} + 1/2G(H_2) - G(OOH*)$

$= \{4.92 + E_{H2O} + ZPE_{H2O} – TS_{H2O}) -2(E_{H2} + ZPE_{H2} - TS_{H2})\} +1/2(E_{H2} + ZPE_{H2} - TS_{H2}) - (E_{OOH*} + ZPE_{OOH*} - TS_{OOH*})$

Or $= 4.92 + 2(E_{H2O} + ZPE_{H2O} – TS_{H2O}) - 3/2(E_{H2} + ZPE_{H2} - TS_{H2}) - (E_{OOH*} + ZPE_{OOH*} - TS_{OOH*})$

where the asterisk (*) refers to the catalyst and active adsorption site on the catalyst. Moreover, the overview of the error arising from the computing of the O_2 molecule with DFT methods can be escaped by fixing the overall Gibbs reaction energy, Gibbs free energy of O_2 to the experimental value of 4.92 eV. Hence, an ideal catalyst with overpotential (η) = 0 V would perfectly allocate the overall change in Gibb's energy throughout the four elementary OER steps i.e., $\Delta G_{a–d}$ = -1.23 eV.

Using the overpotential (η), OER catalytic activity can be further justified if all four steps have different Gibbs free energy values. The overpotential can be calculated using the following equation.

$$\eta^{OER} = \max \{\Delta G_a, \Delta G_b, \Delta G_c, \Delta G_{4d}\}/e - 1.23 \quad (S6)$$

To construct the models of Ir doped and CoIr co-doped on the PdCu (110) nanocluster, four layers of 4×4 supercells of PdCu (110) are sliced. Ir and Co atoms randomly replace several Pd and Cu

atoms in the PdCu (110) nanocluster. In structural optimization calculations, the bottom layer of atoms was fixed and other layers of atoms were allowed to relax.

D-band

To investigate the effects of multimetallic alloy nanocrystals, the d-band center of the added Ir metal atoms on the nanocrystals is calculated by using the following equation:

$$\varepsilon d = \frac{\int_{-\infty}^{\infty} n_d(\varepsilon)\varepsilon d\varepsilon}{\int_{-\infty}^{\infty} n_d(\varepsilon) d\varepsilon}$$

Where the n_d signifies the d states of the metal atoms (M), ε shows the Kohn–Sham eigenvalues ∞ and represents the Fermi energy. It has been reported that the d-band center of the M atoms is closely associated with the catalytic activity

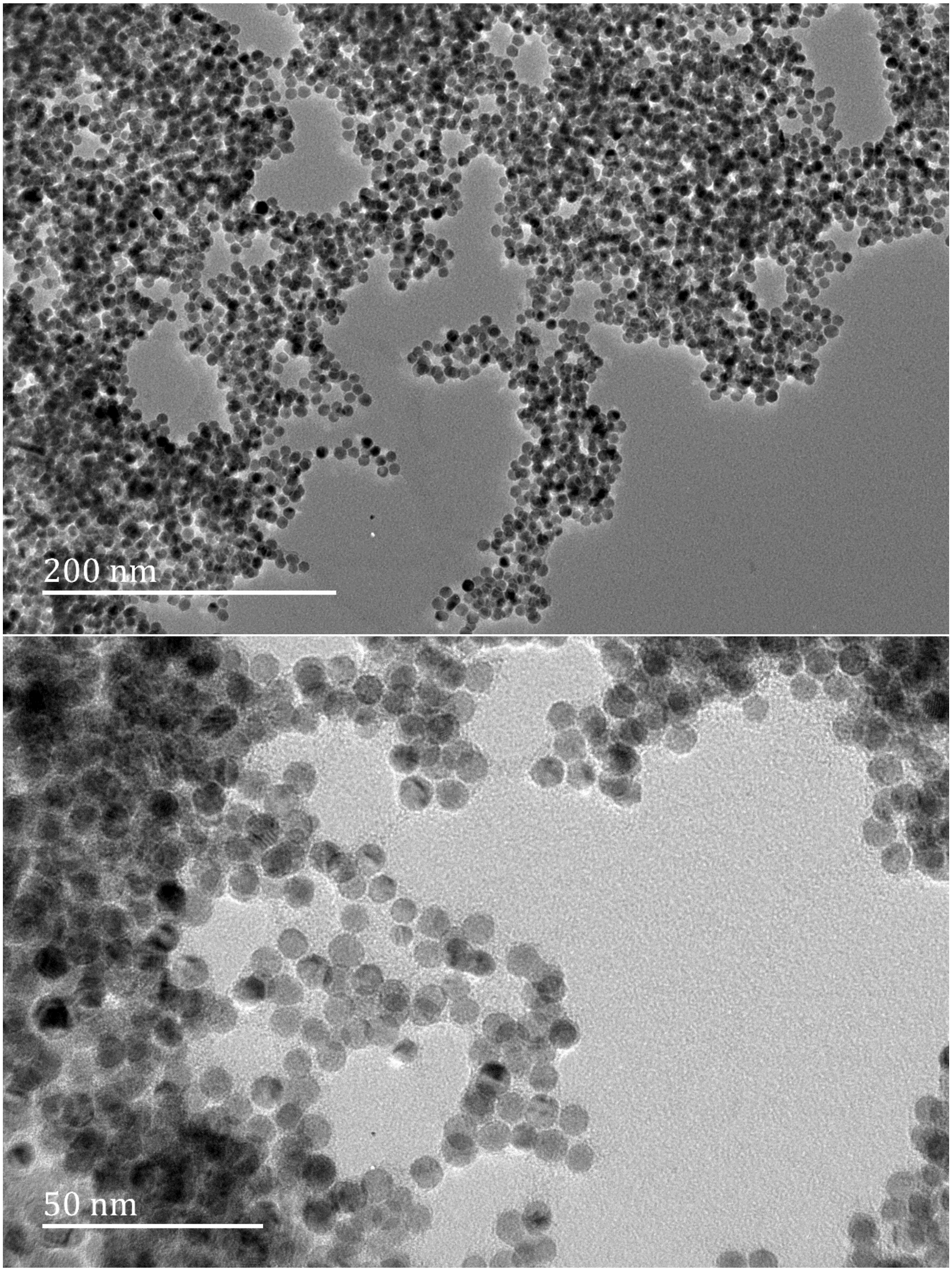

Figure S1. TEM images of PdCuIrCo nanoplates at different magnification.

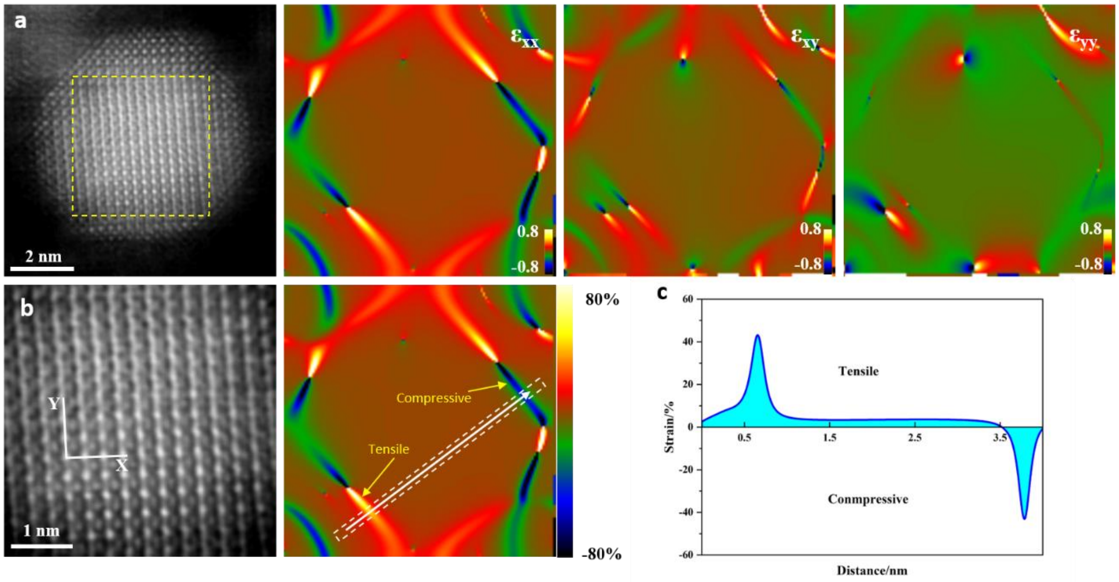

Figure S 2. (a) Abreaction-corrected HAADF-STEM image of PdCuIrCo nanoplate, (b) image taken from the marked box in (a) and the surface strain mapping for marked box with in-plan strain tensors εxx, εyy, εxy, through GPA, the color regions ranging from green to dark blue denote the compressive strain, while the regions from red to bright yellow represent the tensile strain.[10,11] Note that the signal from regions outside the areas marked by two white quadrilaterals is the noise caused by blurring the STEM image.[12,13] (c) Strain distribution along the white arrow in panel εxx for PdCuIrCo nanoplate. Results shows that nanoplates are mostly dominated by the tensile-strain. [10,11,12,13]

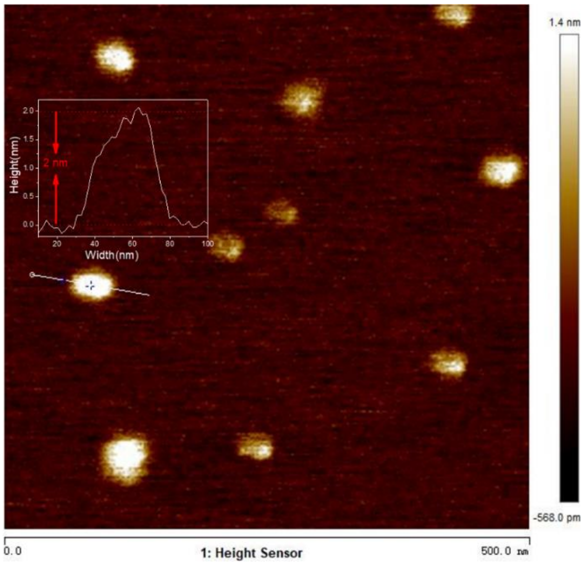

Figure S3. TEM topography showing the flat-top morphology of PdCuIrCo nanoplates with a line profile of the nanoplate. The thickness of a nanoplate is about 2nm.

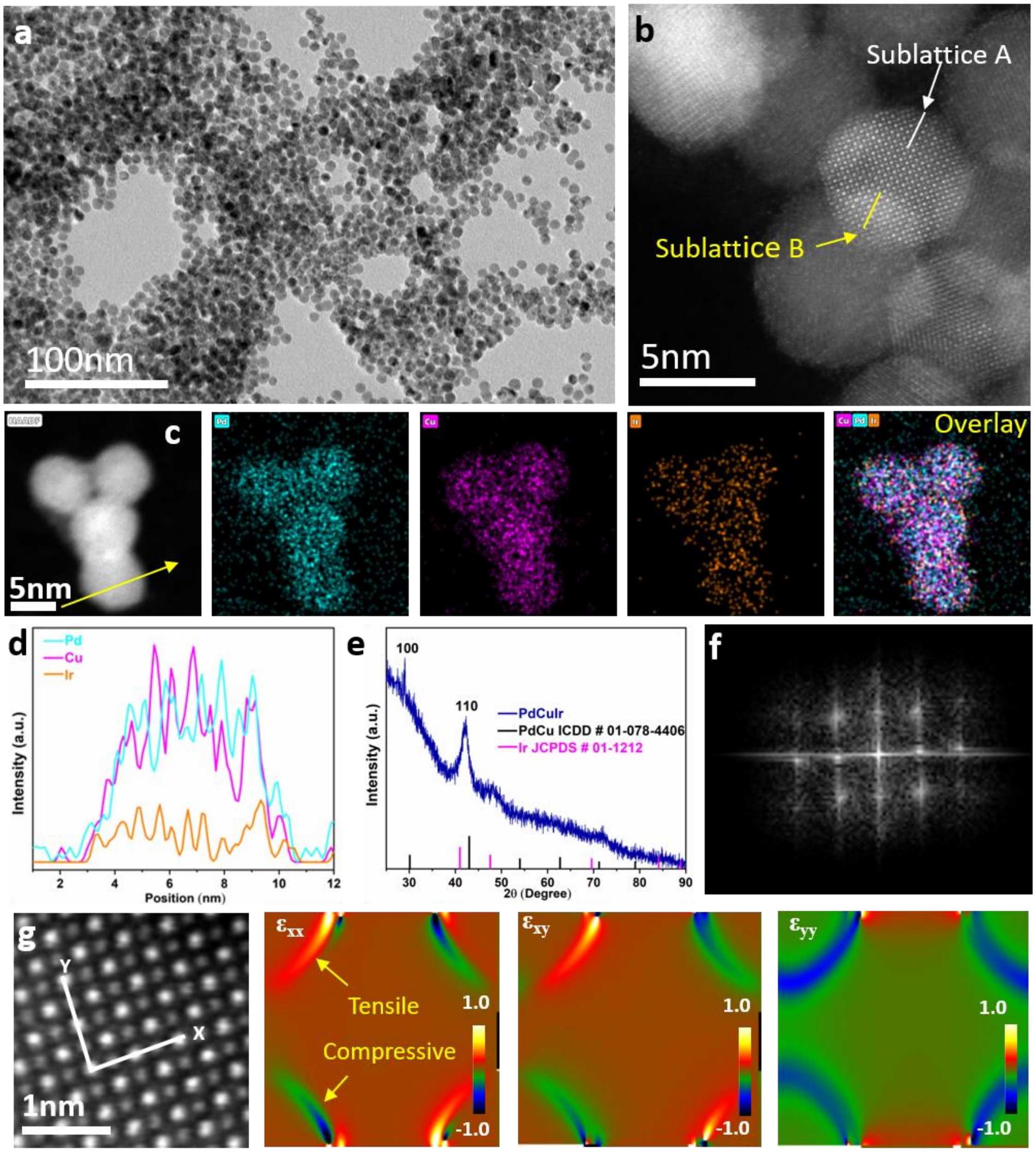

Figure S 4. Structural and surface strain characterizations of the PdCuIr nanoplates: (a) TEM image, (b) HAADF-STEM image, (c) HAADF-STEM image and the corresponding EDS elemental mapping, (d) line-scanning profiles across the yellow arrow shown in the image of figure c, (e) PXRD pattern, (f) FFT pattern of PdCuIr nanoplate and (g) maps of the in-plan strain tensors εxx, εyy, εxy, processed via GPA taken from the yellow frame area in figure e, (the color regions ranging from green to dark blue denote the compressive strain, while the regions from red to bright yellow represent the tensile strain).

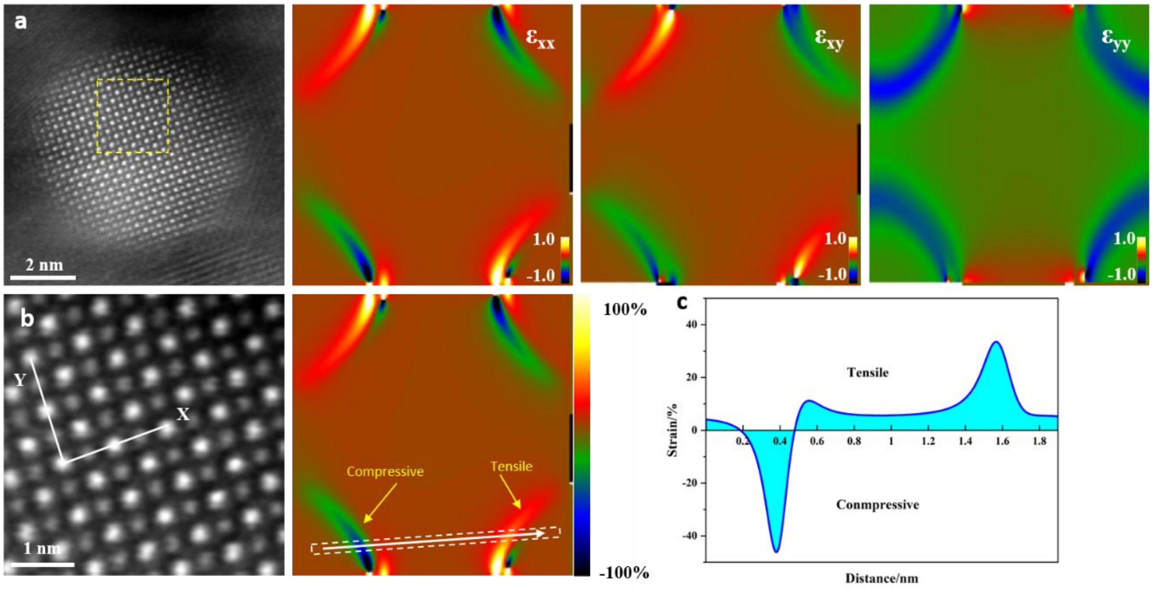

Figure S 5. (a) Abreaction-corrected HAADF-STEM image of PdCuIr nanoplate, (b) image taken from the marked box in (a) and the surface strain mapping for marked box with in-plan strain tensors εxx, εyy, εxy, through GPA, the color regions ranging from green to dark blue denote the compressive strain, while the regions from red to bright yellow represent the tensile strain.[10,11] Note that the signal from regions outside the areas marked by two white quadrilaterals is the noise caused by blurring the STEM image.[12,13] (c) Strain distribution along the white arrow in panel εxx for PdCuIr nanoplate. Results shows that nanoplates are mostly dominated by the tensile-strain. [10,11,12,1]

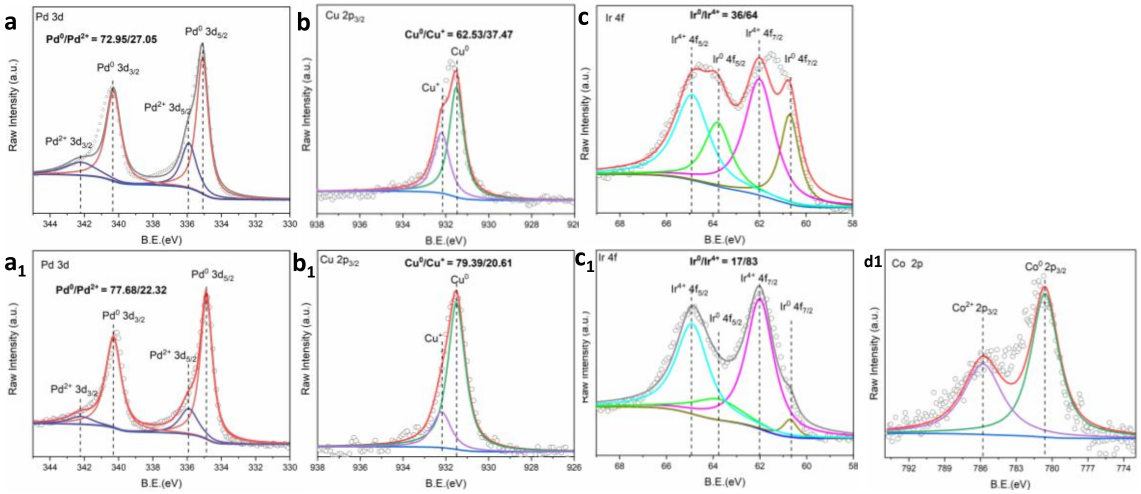

Figure S6. XPS patterns of Pd 3d from (a) PdCuIr NPLs and (a1) PdCuIrCo NPLs, the Cu 2p from (b) PdCuIr NPLs and (b1) PdCuIrCo NPLs, the Ir 4f from (c) PdCuIr NPLs and (c1) PdCuIrCo NPLs and the Co 2p from (d1) PdCuIrCo NPLs.

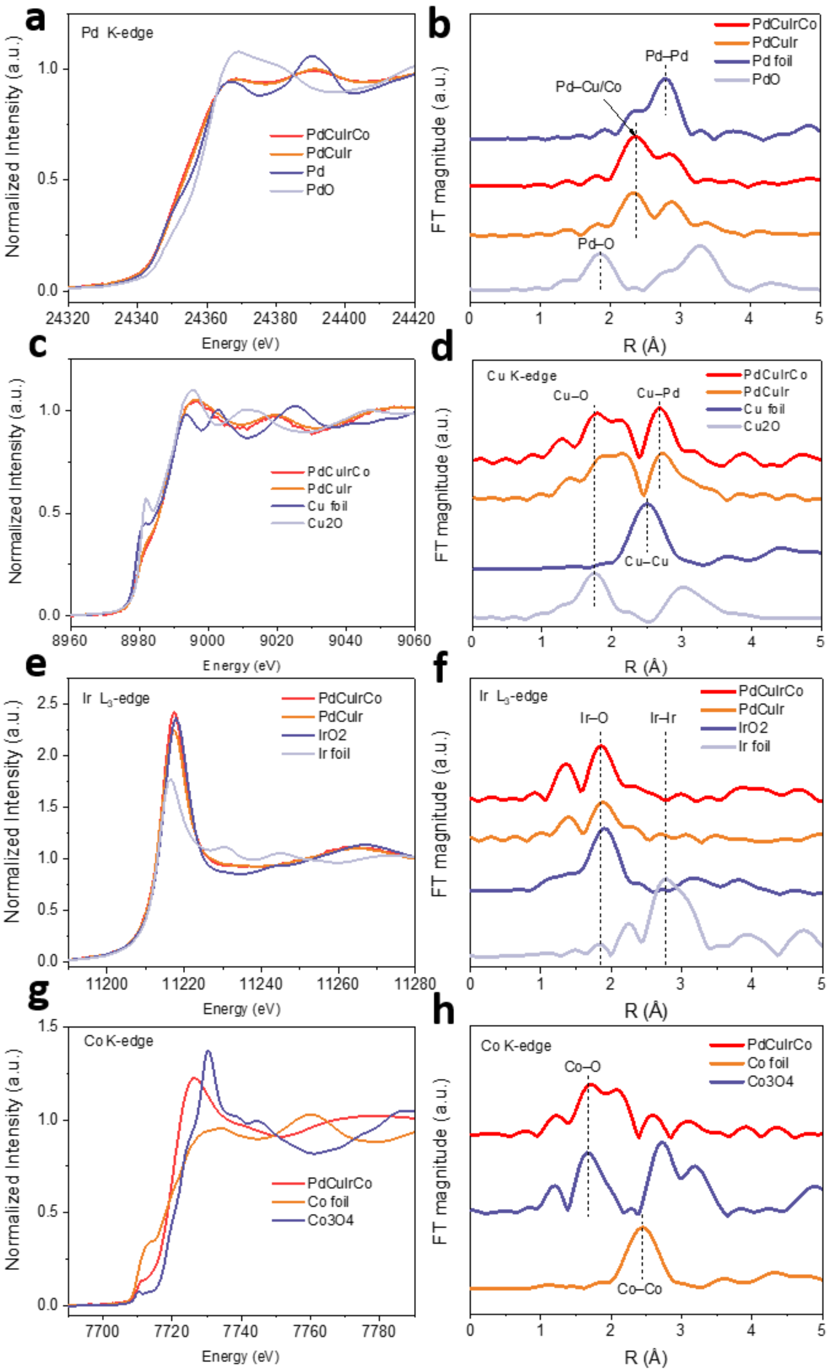

Figure S 7. (a, c, e, g) X-ray absorption near-edge structure (XANES), and (b, d, f, h) extended X-ray absorption fine structure (EXAFS) spectra of as-prepared PdCuIr and PdCuIrCo nanoplates and their refence samples.

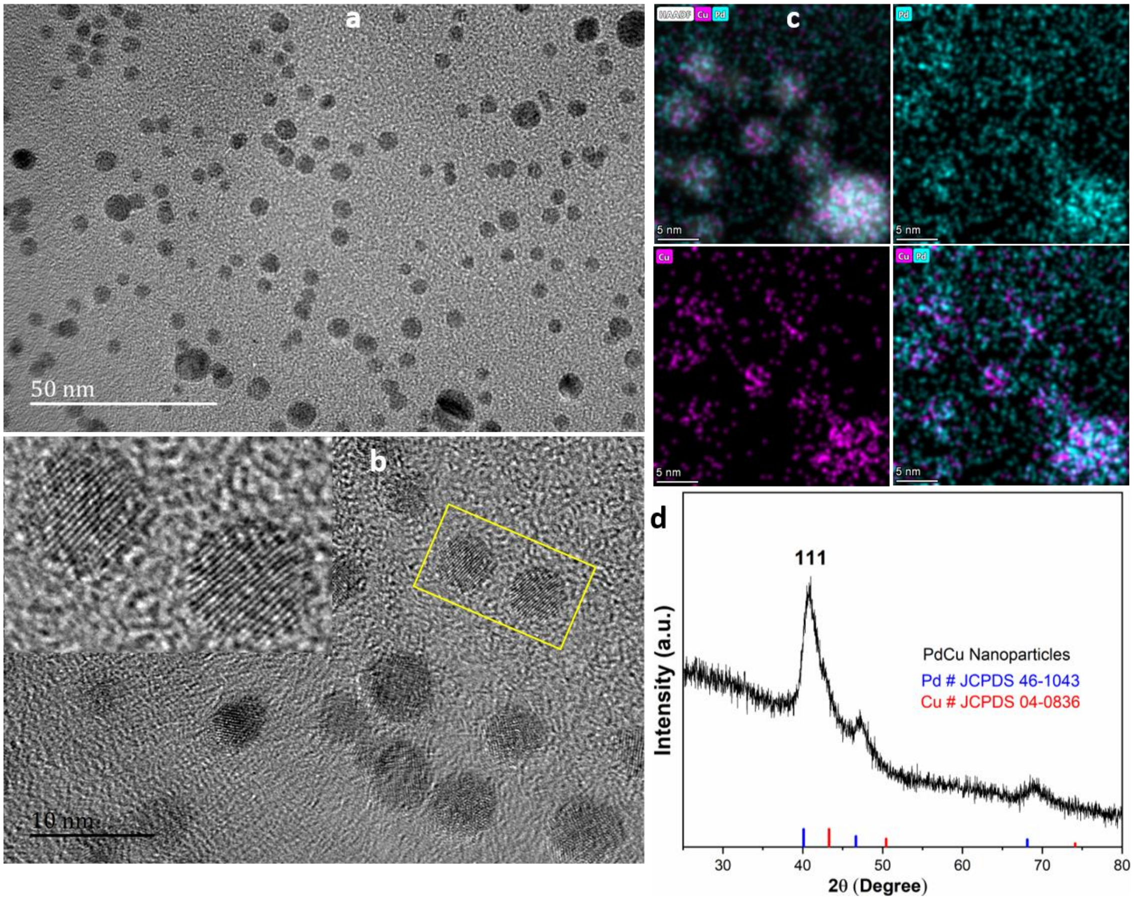

Figure S 8. Structural characterizations of the PdCu nanoparticles: (a) TEM image, (b) HRTEM image, (c) HAADF-STEM image and the corresponding EDS elemental mapping, (d) PXRD pattern of PdCu nanoparticles.

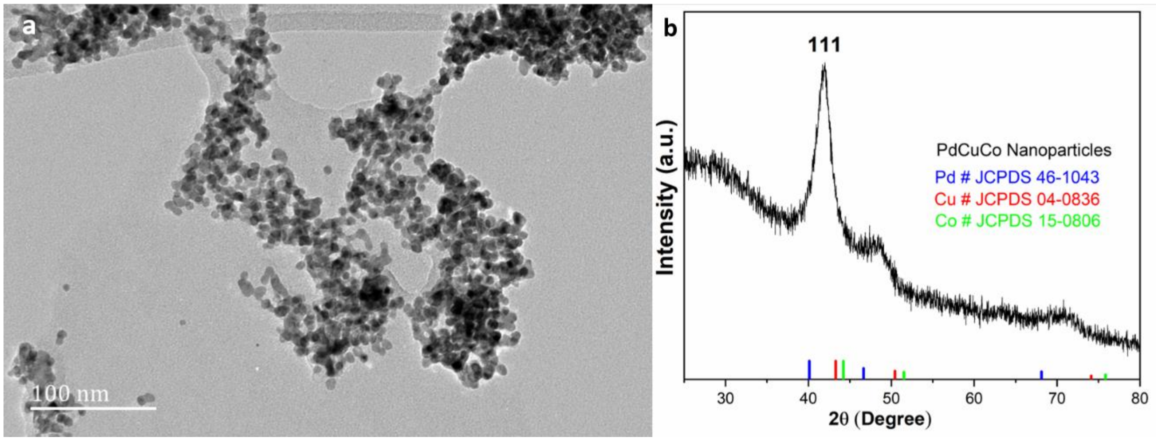

Figure S 9. Structural characterizations of the PdCuCo nanoparticles: (a) TEM image (b) PXRD pattern of PdCuCo nanoparticles.

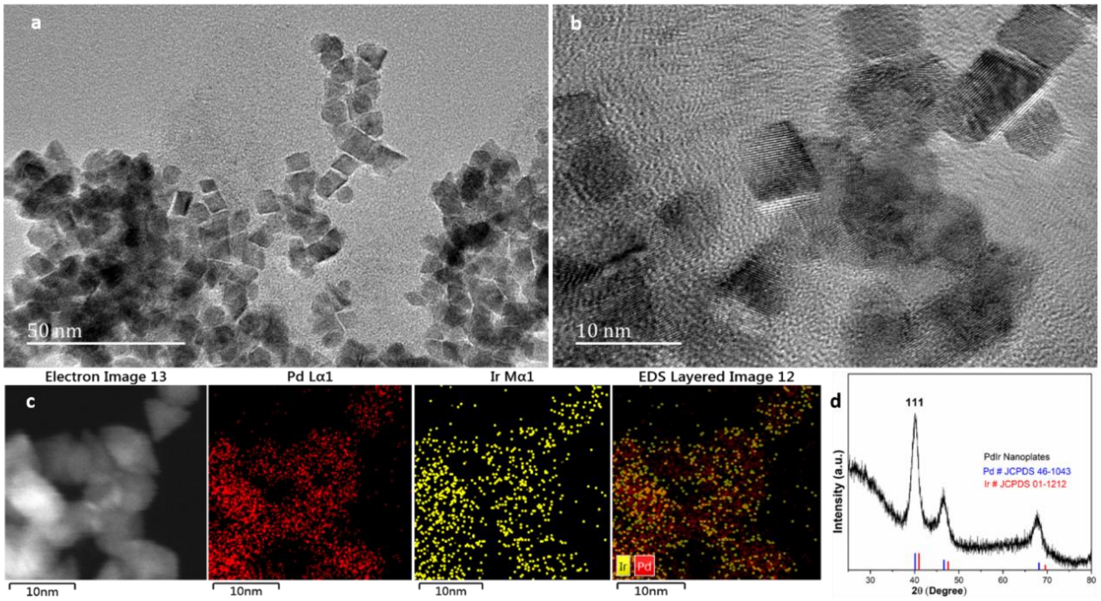

Figure S 10. Structural characterizations of the PdIr nanoplates: (a) TEM image, (b) HRTEM image, (c) HAADF-STEM image and the corresponding EDS elemental mapping, (d) PXRD pattern of PdIr nanoplates.

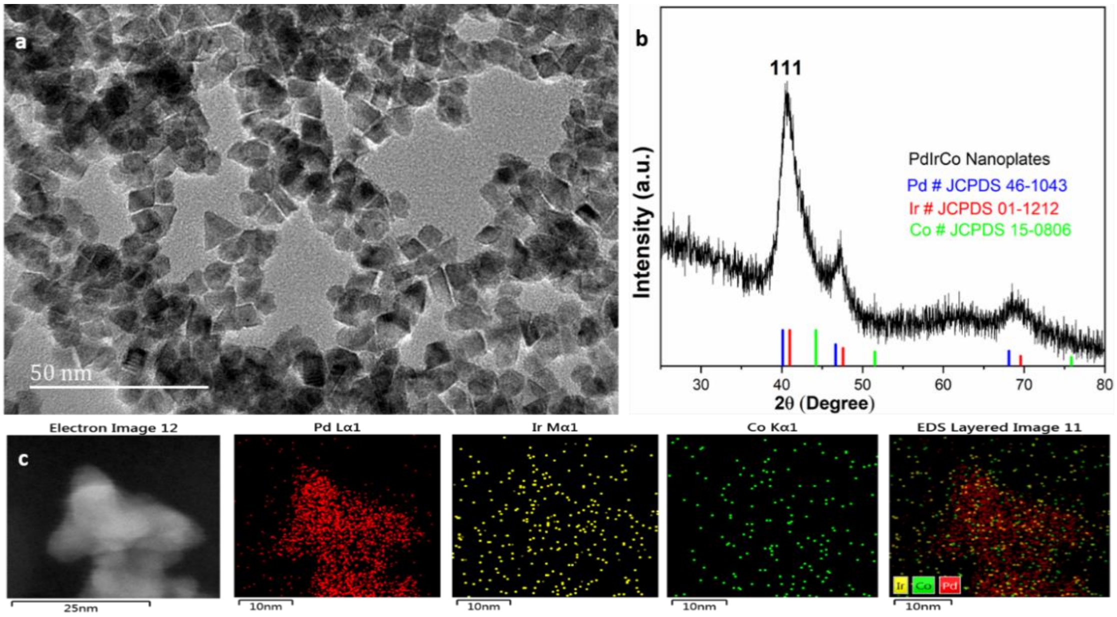

Figure S11. Structural characterizations of the PdIrCo nanoplates: (a) TEM image, (b) PXRD pattern, (c) HAADF-STEM image and the corresponding EDS elemental mapping of PdIrCo nanoplates.

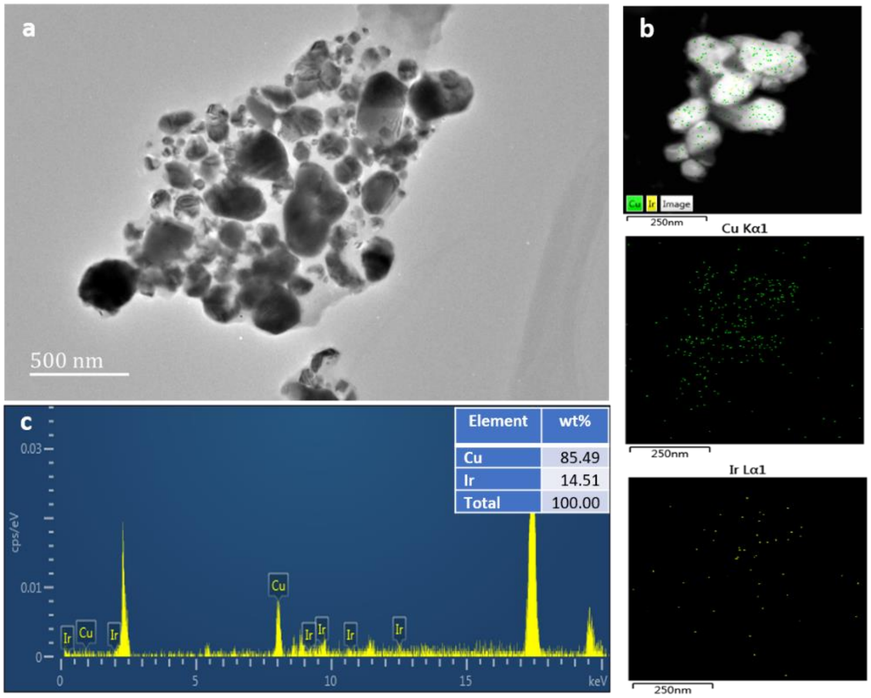

Figure S 12. Morphological characterizations of the CuIr nanocrystals: (a) TEM image, (b) HAADF-STEM image and the corresponding EDS elemental mapping of CuIr nanocrystals and (c) TEM-EDX spectrum image of CuIr nanocrystals.

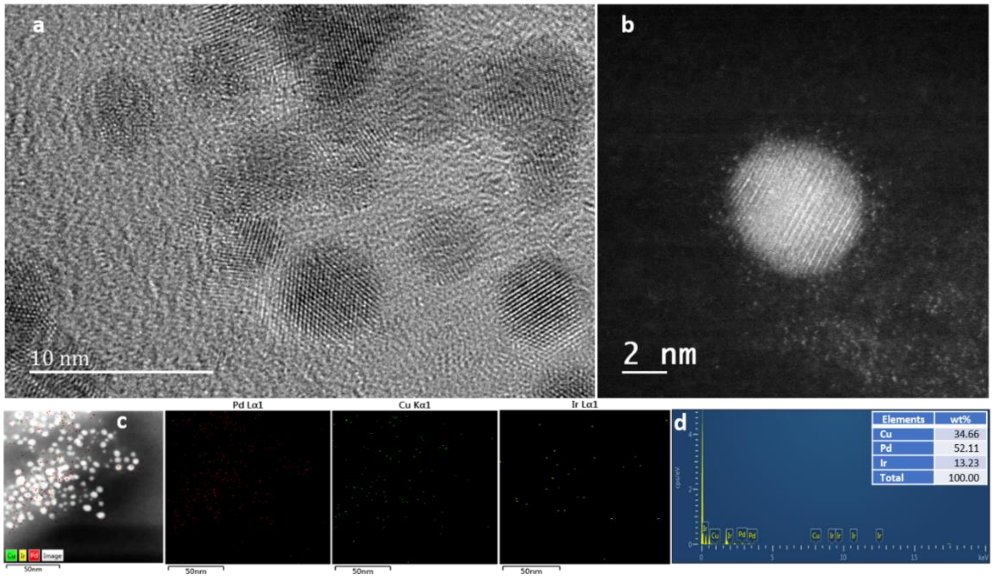

Figure S 13. PdCuIr nanocrystals synthesized at 30 min reaction time: (a) TEM image, (b) HAADF-STEM image, (c) HAADF-STEM image and the corresponding EDS elemental mapping of CuIr nanocrystals and (d) TEM-EDX spectrum image of PdCuIr nanocrystals.

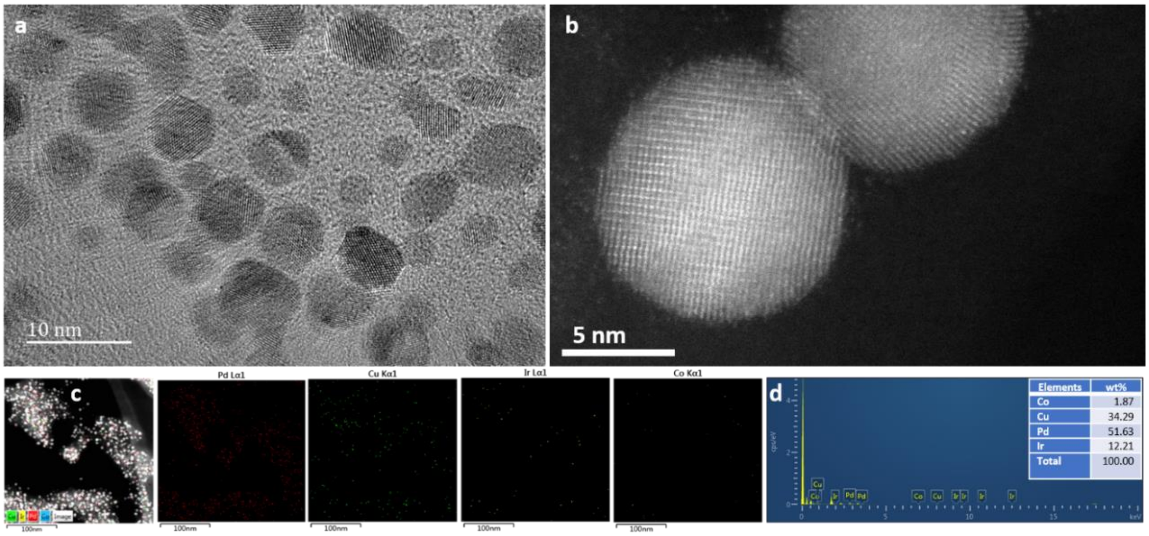

Figure S 14. PdCuIrCo nanocrystals synthesized at 30 min reaction time: (a) TEM image, (b) HAADF-STEM image, (c) HAADF-STEM image and the corresponding EDS elemental mapping of PdCuIrCo nanocrystals and (d) TEM-EDX spectrum image of PdCuIrCo nanocrystals.

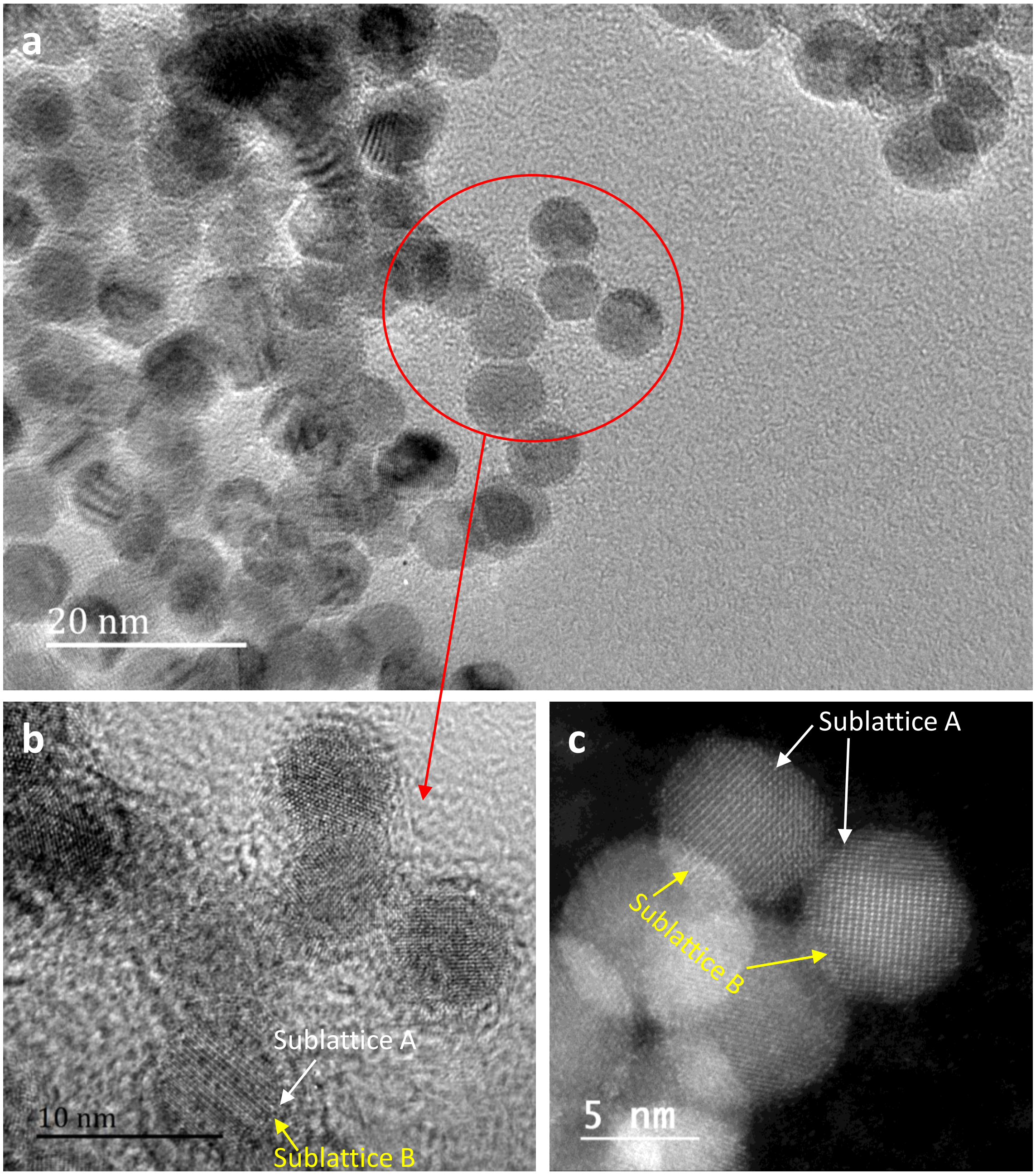

Figure S 15. PdCuIrCo nanoplates synthesized at 60 min reaction time: (a) TEM image, (b) HRTEM image, (c) HAADF-STEM image of PdCuIrCo nanoplates.

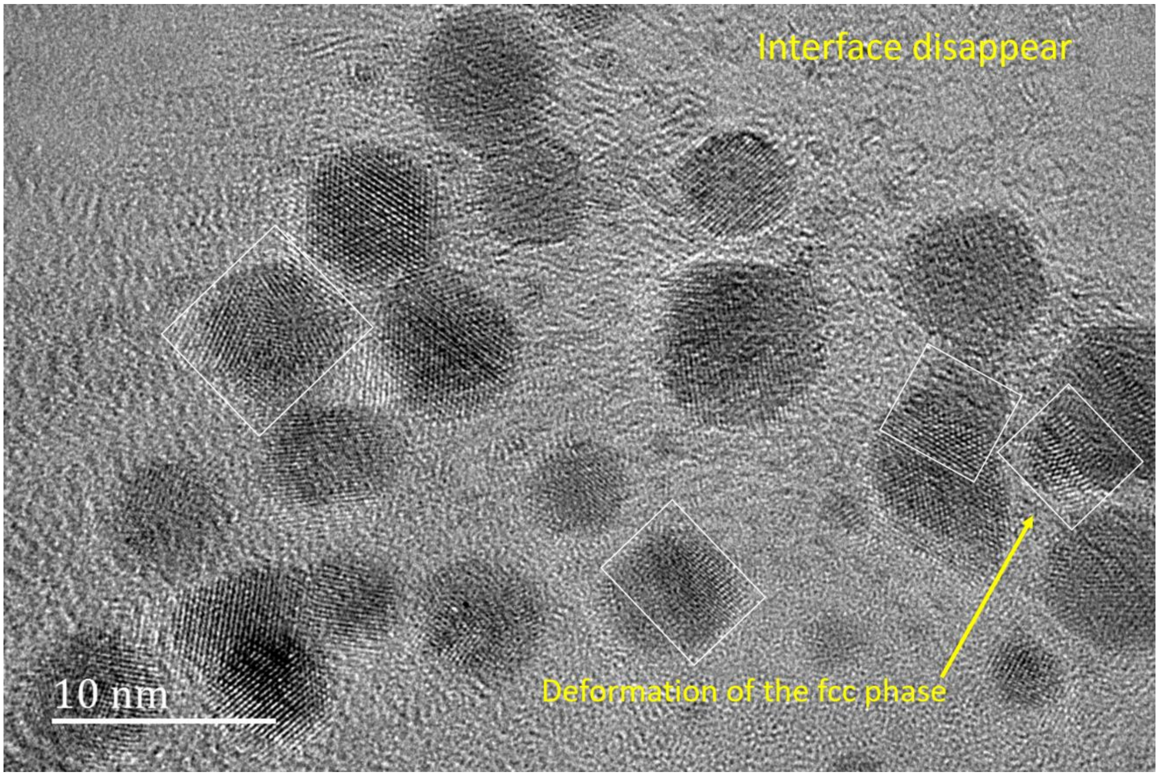

Figure S 16. HRTEM image of PdCuIr nanocrystals synthesized at 15-20 min reaction time.

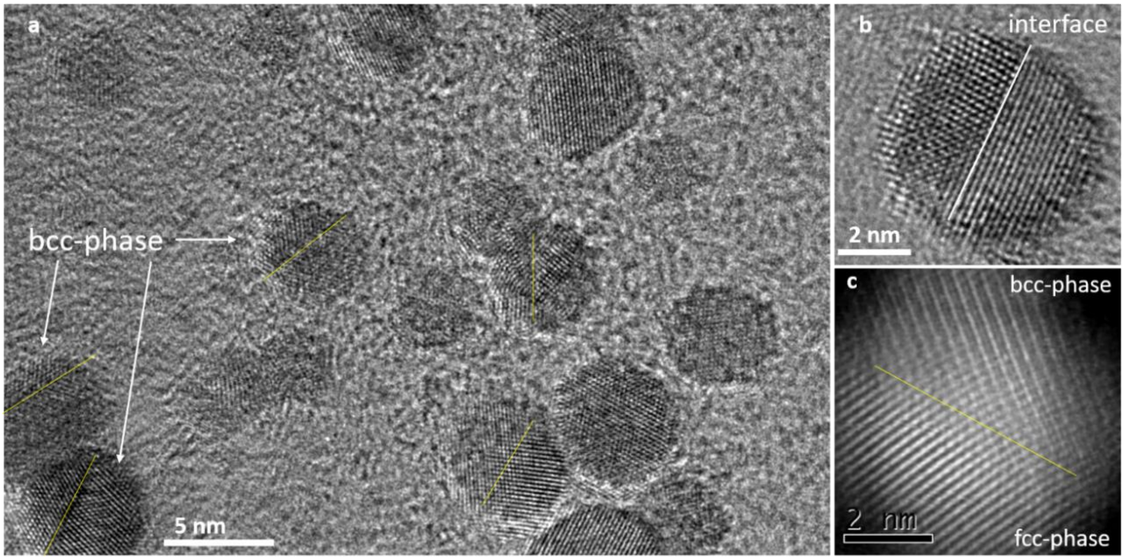

Figure S 17. Characterizations of the PdCuIr nanocrystals synthesized at 10-15 min reaction time: (a,b) HRTEM images, (c) HAADF-STEM image.

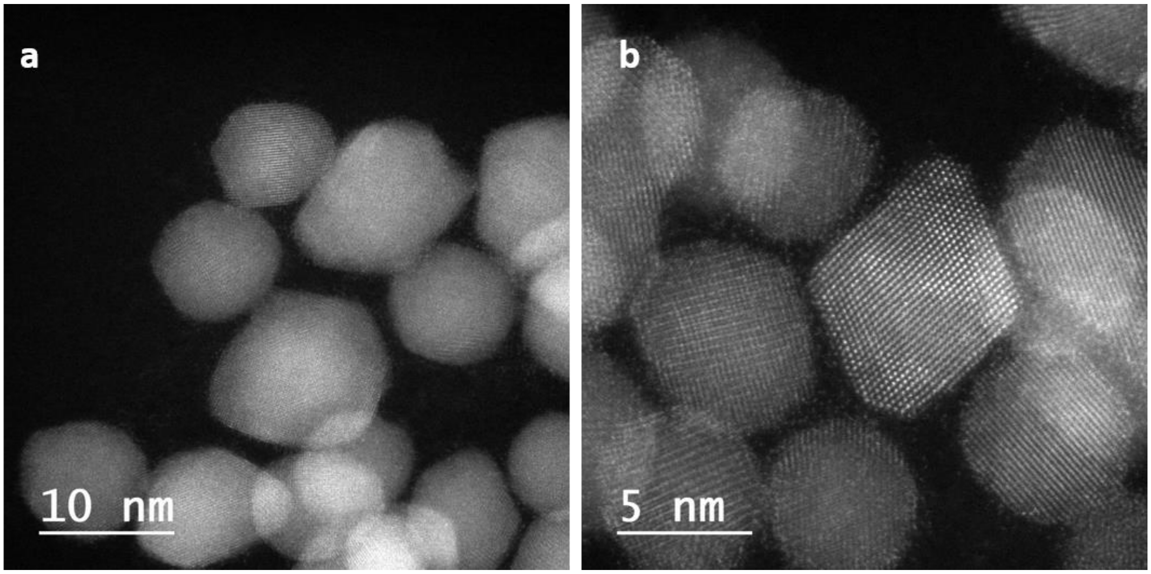

Figure S 18. Characterization of the PdCuIrCo nanocrystals synthesized at (a) low and (b) high concentration of Ir.

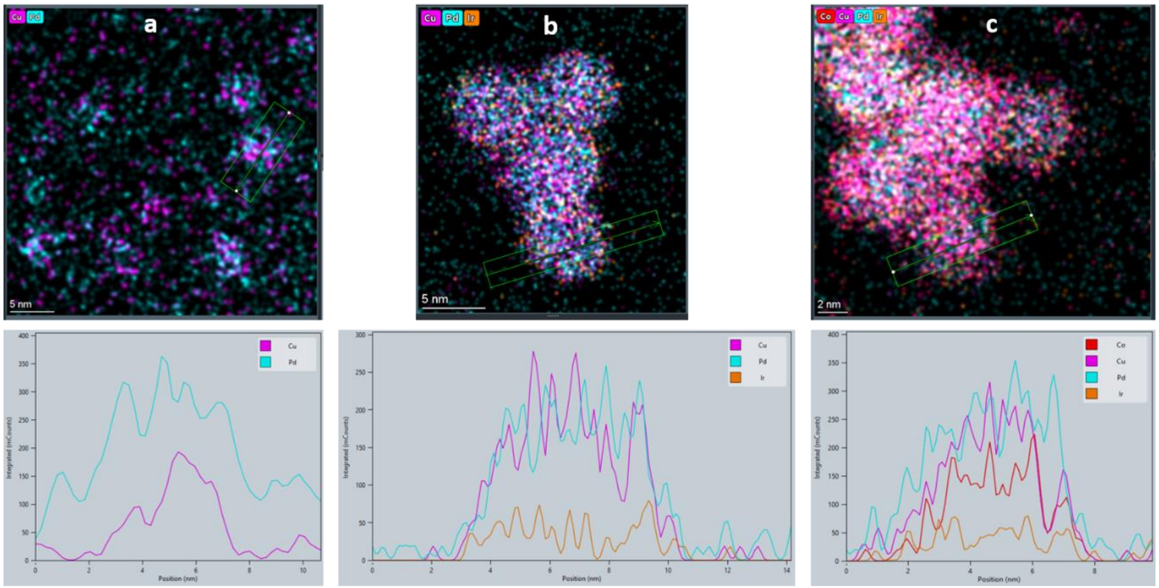

Figure S 19. EDS line scan composition profiles analysis of nanocrystals: (a) PdCu nanocrystals, (b) PdCuIr nanoplates, (c) PdCuIrCo nanoplates.

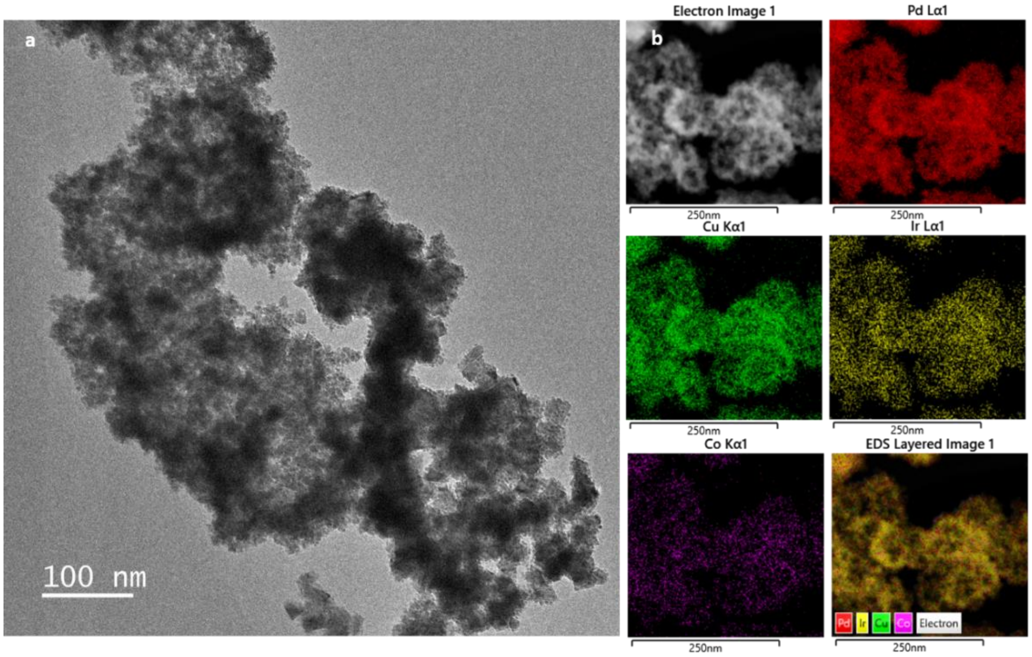

Figure S 20. Structural characterizations of the PdCuIrCo nanocrystals synthesized without gel: (a) TEM image, (b) HAADF-STEM image and the corresponding EDS elemental mapping of PdCuIrCo nanocrystals.

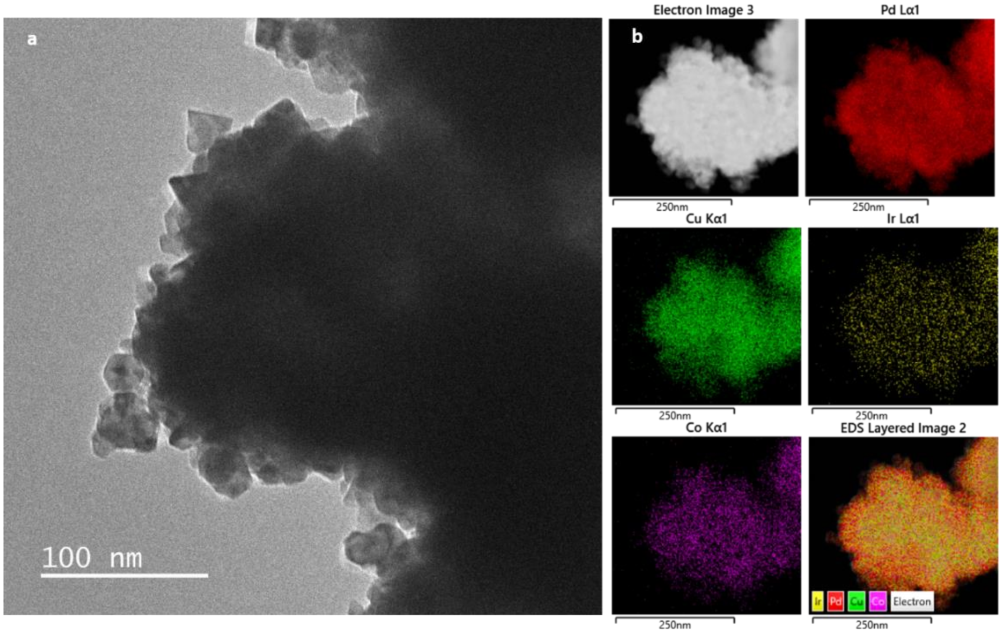

Figure S 21. Structural characterizations of the PdCuIrCo nanocrystals synthesized by mixing all the gel components together with precursors in one-step: (a) TEM image, (b) HAADF-STEM image and the corresponding EDS elemental mapping of PdCuIrCo nanocrystals.

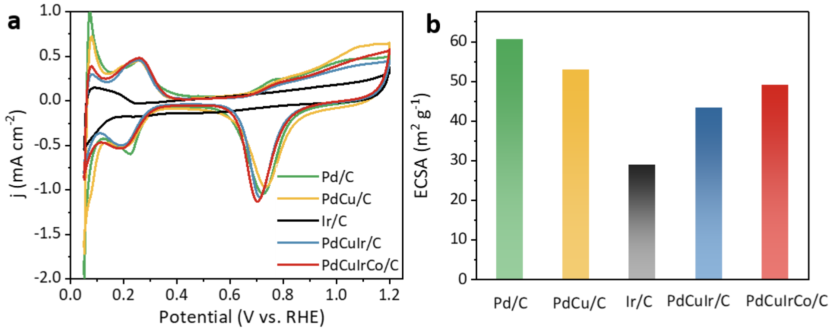

Figure S 22. (a) CV curves recorded from the PdCuIrCo/C, PdCuIr/C, PdCu/C and commercial 20% Ir/C and 20% Pd/C, and (b) their corresponding ECSAs at an Pd+Ir loading of 10.2 μg·cm^{-2} on RDE.

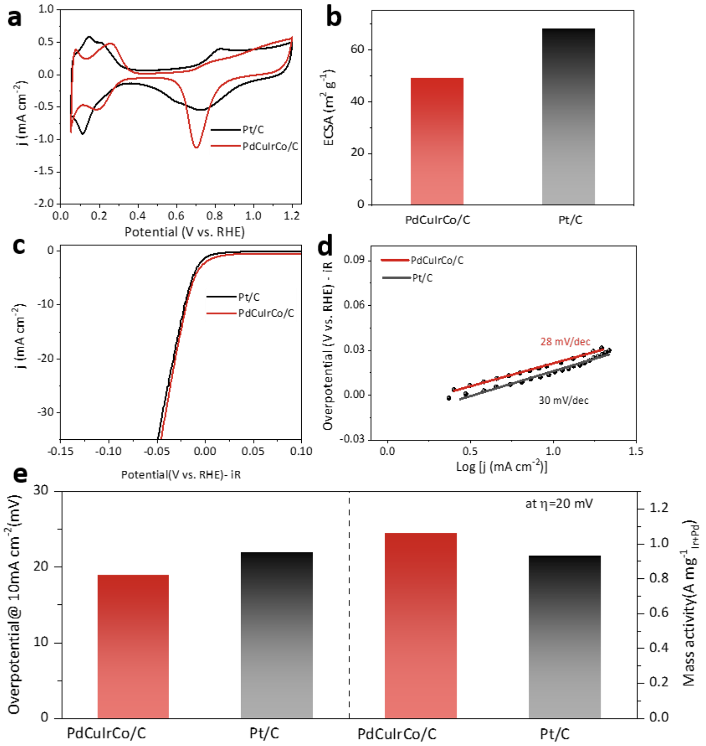

Figure S23. (a) CV curves recorded from the PdCuIrCo/C and commercial Pt/C. (b) their corresponding ECSAs at an Pd+Ir or Pt loading of 10.2 μg·cm^{-2} on RDE. (c) The HER polarization curves recorded with a linear scan of potential at 5 mV s^{-1} from the PdCuIrCo/C and Pt/C. (d) Tafel plot of PdCuIrCo/C and Pt/C electrocatalysts. (e) The overpotential at a current density of 10 mAcm^{-2} (left) and mass activity at an overpotential of 20 mV (vs. RHE) of PdCuIrCo/C and Pt/C electrocatalysts (right).

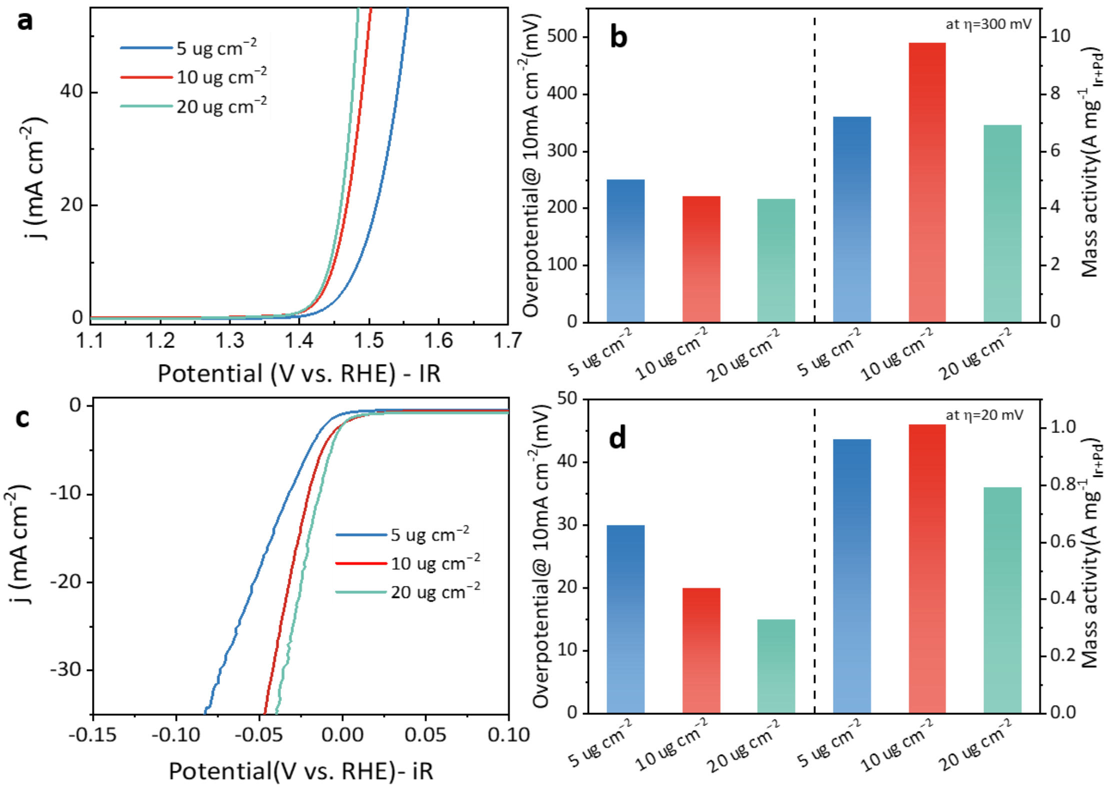

Figure S24. (a) The OER polarization curves recorded with a linear scan of potential at 5 mV s^{-1} from the PdCuIrCo/C with different loading. (b) The overpotential at a current density of 10 $mAcm^{-2}$ (left) and mass activity at an overpotential of 300 mV (vs. RHE) of PdCuIrCo/C with different loading(right). (c) The HER polarization of PdCuIrCo/C with different loading curves recorded with a linear scan of potential at 5 mV s^{-1}. (d) Tafel plot of PdCuIrCo/C with different loading.

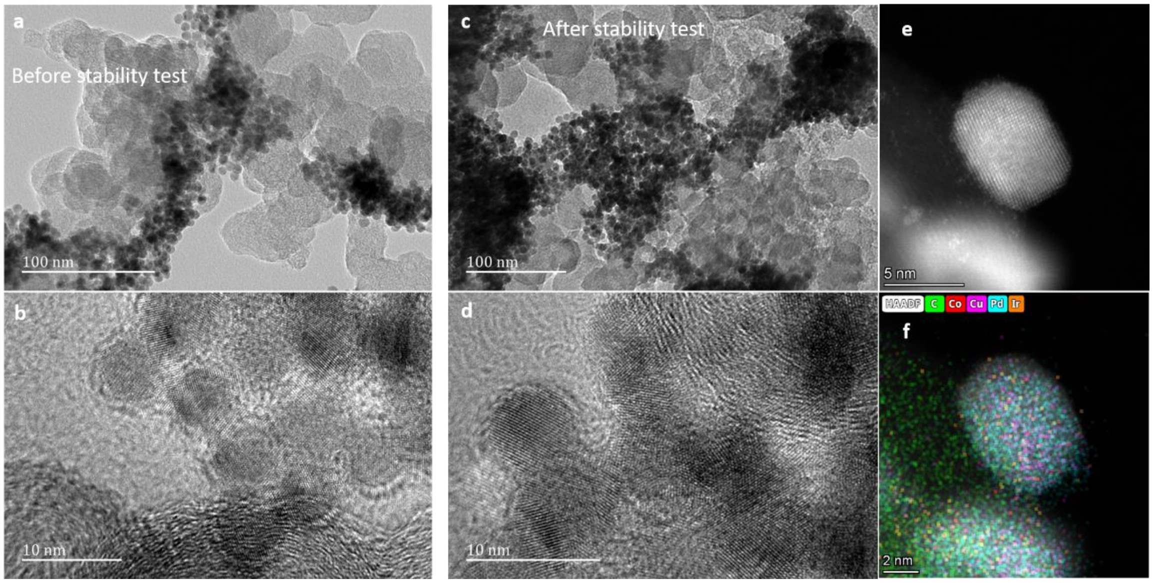

Figure S 25. (a) TEM image, (b) HRTEM image of PdCuIrCo/C before stability test, (c) TEM image, (d) HRTEM image, (e) HAADF-STEM image, and (f) Elemental STEM-HAADF image of PdCuIrCo/C after stability test.

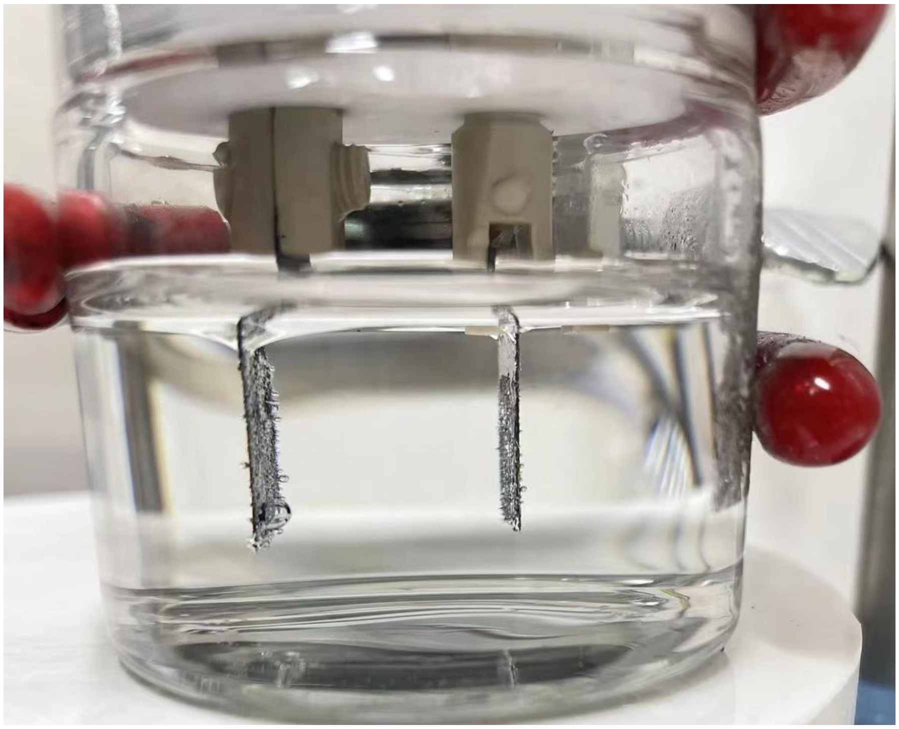

Figure S26. (a) The electrolyzer in a working condition.

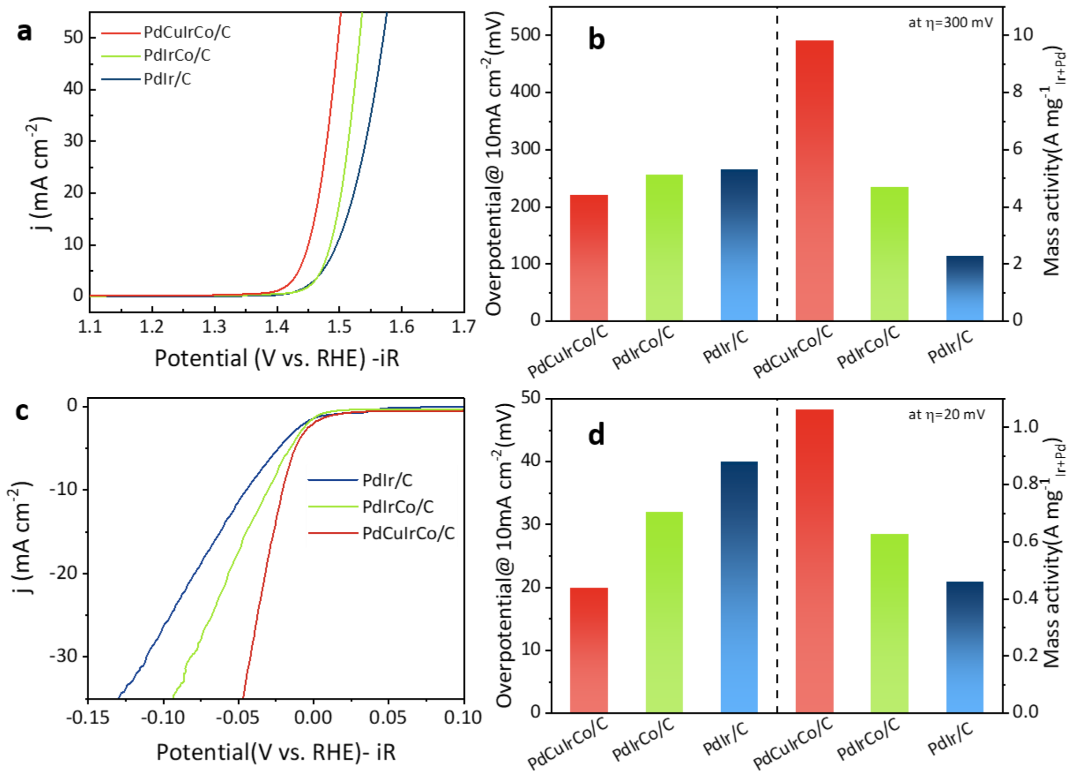

Figure S27. (a) The OER polarization curves recorded with a linear scan of potential at 5 mV s^{-1} from the PdCuIrCo/C, PdIrCo/C and PdIr/C , respectively. (b) The overpotential at a current density of 10 $mAcm^{-2}$ (left) and mass activity at an overpotential of 300 mV (vs. RHE) of different electrocatalysts (right). (c) The HER polarization of different electrocatalysts curves recorded with a linear scan of potential at 5 mV s^{-1}. (d) The overpotential (left) and mass activity(right).

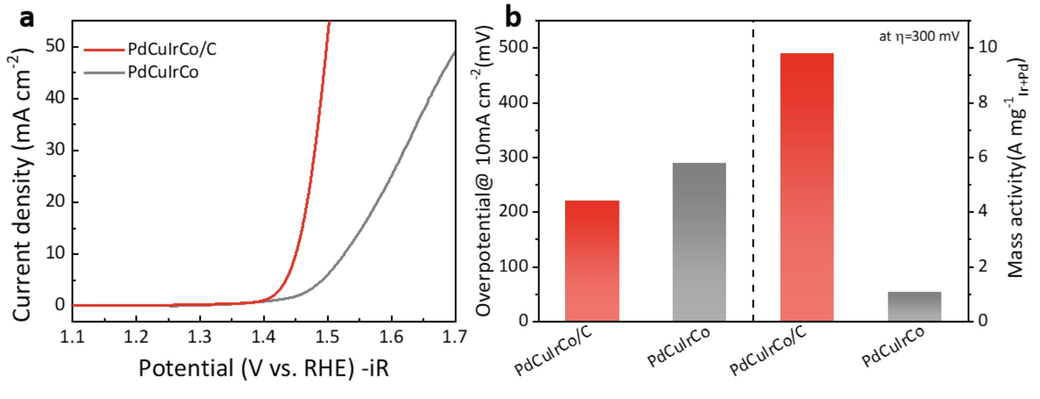

Figure S28. (a) The OER polarization curves recorded with a linear scan of potential at 5 mV s^{-1} from the PdCuIrCo (without carbon) and PdCuIrCo/C , respectively. (b) The overpotential at a current density of 10 $mAcm^{-2}$ (left) and mass activity at an overpotential of 300 mV (vs. RHE) of different electrocatalysts (right).

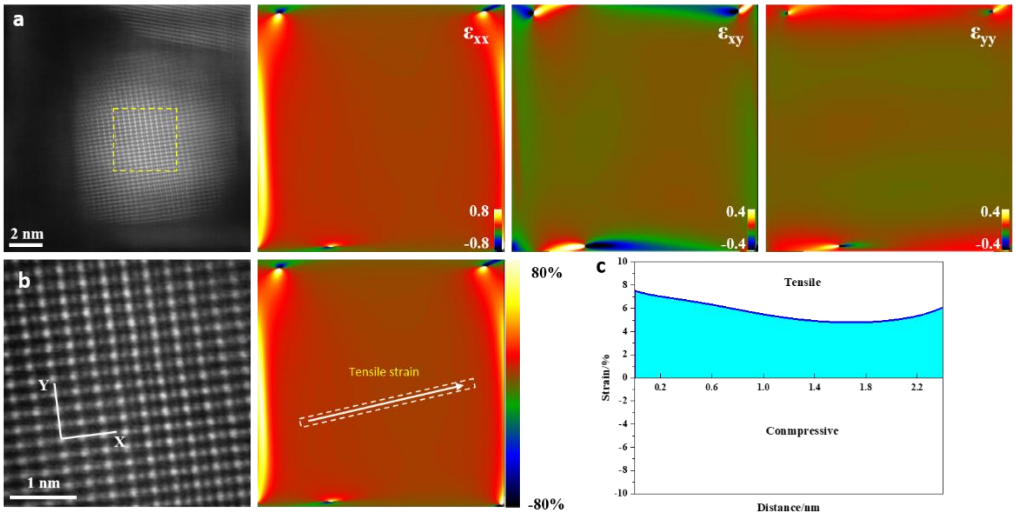

Figure S 29. (a) Abreaction-corrected HAADF-STEM image of PdCuIrCo nanoplate, (b) image taken from the marked box in (a) and the surface strain mapping for marked box with in-plan strain tensors εxx, εyy, εxy, through GPA, the color regions ranging from green to dark blue denote the compressive strain, while the regions from red to bright yellow represent the tensile strain.[10,11] Note that the signal from regions outside the areas marked by two white quadrilaterals is the noise caused by blurring the STEM image.[12,13] (c) Strain distribution along the white arrow in panel εxx for PdCuIrCo nanoplate. Results shows that nanoplates are mostly dominated by the tensile-strain.[10,11,12,13]

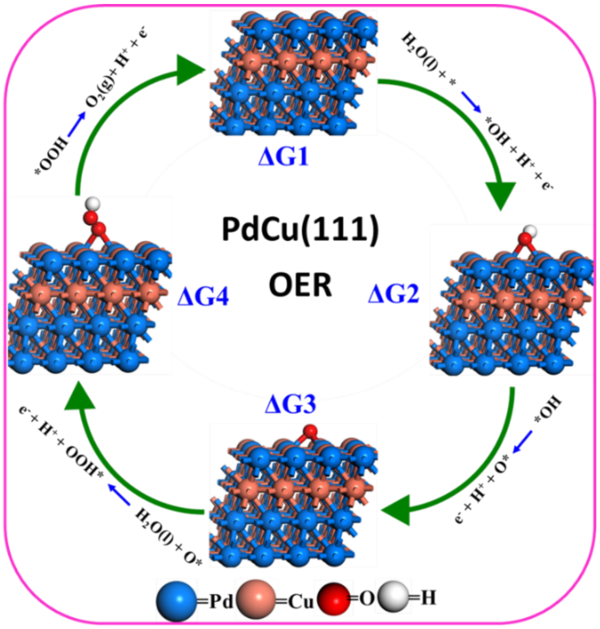

Figure S30. Schematic of the 4e⁻ OER pathway of the active site over the PdCu (111) NC with the optimized configurations for intermediates.

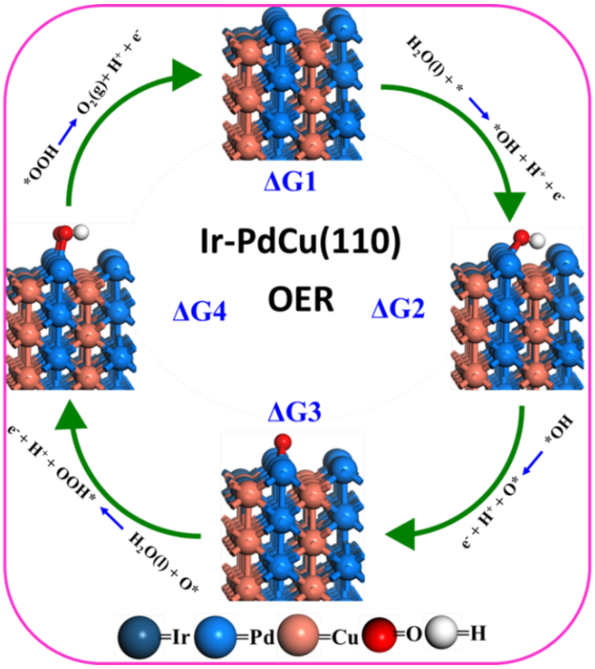

Figure S31. Schematic of the 4e⁻ OER pathway of the active site over the PdCuIr (110) NC with the optimized configurations for intermediates.

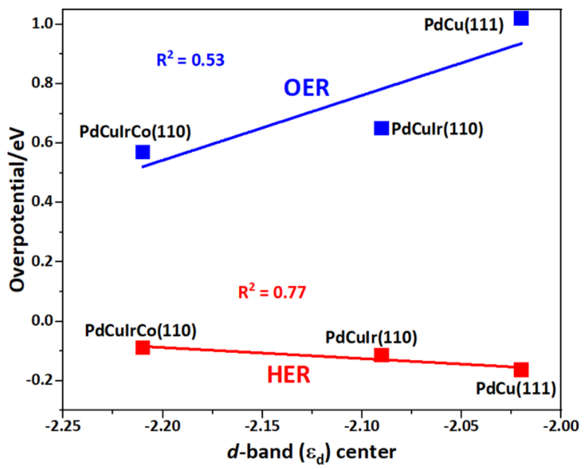

Figure S 32. The relationship between the overpotential and d-band.

Table S1. The wt.% of Pd, Cu and Ir measured by ICP-MS in the PdCuIr samples at different reaction time.

sample	Ir wt%	Cu wt%	Ir wt%
PdCuIr NCs at 5 min	84.81	12.92	2.27
PdCuIr NCs at 10 min	75.21	20.11	4.68
PdCuIr NCs at 20 min	63.31	29.56	7.13
PdCuIr NCs at 30 min	50.11	37.96	12.93
PdCuIr NCs at 180 min	42.21	42.32	15.27

Table S2. The wt.% of Pd, Cu, Ir and Co measured by ICP-MS in the PdCuIrCo samples at different reaction time.

Catalyst	Ir wt%	Cu wt%	Ir wt%	Co wt%
PdCuIrCo NCs at 30 min	50.21	35.26	11.82	2.71
PdCuIr NCs at 180 min	31.42	31.00	18.10	19.48

Table S3. The wt.% of elements measured by ICP-MS for the Pd, Cu, Ir and Co contents in the catalysts.

Catalyst	Pd wt%	Cu wt%	Ir wt%	Co wt%
PdCu catalyst	70.0	30.0	0	0
PdCuIr catalyst	42.21	42.32	15.27	0
PdCuIrCo catalyst	31.42	31.00	18.10	19.48
PdCuIrCo catalyst after stability	32.91	30.20	18.00	18.89

Table S4. Comparisons of OER activity of the as-prepared and commercial Ir/C and Pd/C catalysts.

Catalyst	Overpotentials (η) at 10 mA cm^{-2} (mV)	Tafel (mV dec^{-1})	OER-Mass activity (A mg_{Pd+Ir}^{-1}) at overpotential 300 (mV)	ECSA(m^2 g^{-1})
PdCu/C	354	59	0.26 (1.53 V)	52.9
PdCuIr/C	247	52	6 (1.53 V)	43.3
PdCuIrCo/C	221	50	9.8 (1.53 V)	49.1
Ir/C	301	90	0.98 (1.53 V)	28.9
Pd/C	465	145	0.069 (1.53 V)	60.6

Table S5. The OER performance of different noble metal based catalysts in acidic electrolyte solution.

No.	Catalyst	Electrolyte	Overpotential at 10 mA cm^2 (mV)	Mass activity ($mA\ mg^{-1}$)	References
**1**	**PdCuIrCo/C**	**0.1 M $HClO_4$**	**221**	**9.8**	**This work**
2	Cu-Ir	0.05M H_2SO_4	286	NA	J. Mater. Chem. A, 2015, 3, 19669-19673
3	Co-IrCu ONC/C	0.1 m HClO4	293	0.45	Adv. Funct. Mater. 2017, 27, 1604688
4	Ir_3Cu MAs	0.1 m HClO4	298	NA	ACS Energy Lett. 2018, 3, 2038
5	Ir-NR/C	0.05M H_2SO_4	290	0.374	Appl. Catal. B 2020, 279, 119394
6	PdCu/Ir/C	0.05M H_2SO_4	283	1.83	Angew. Chem. Int. Ed. 2021, 60, 8243 – 8250
7	3D Ir	0.1M $HClO_4$	270	NA	Nano. Lett. 2016, 16, 4424-4430
8	Ir nanosheet	0.5M H_2SO_4	240	NA	J. Am. Chem. Soc., 2018, 140, 12434-12441
9	IrNi nanoflower	0.1M $HClO_4$	293	NA	Small Methods, 2019, 1900129
10	Ir_2Sm	0.5M H_2SO_4	275	1.24	Chem. Sci., 2023, 14, 5887-5893
11	RhIr nanoparticle	0.5M H_2SO_4	292	1.17	ACS Nano, 2019, 13, 13225-13234
12	RuCu nanosheet	0.5M H_2SO_4	236	NA	Angew. Chem. Int. Ed., 2019, 131, 14121-14126
13	Ir-Ag nanotube	0.5M H_2SO_4	285	NA	Nano Energy, 2019, 56, 330-337
14	Amorphous Ir nanosheet	0.1M $HClO_4$	255	NA	Nat. Commun., 2019, 10, 4855
15	Co-RuIr	0.1M $HClO_4$	235		Adv. Mater. 2019, 31, 1900510
16	PdCu/Ir	0.1M $HClO_4$	283	1.19	Angew. Chem. Int. Ed., 2021, 60, 8243-8250
17	Ir nanorod	0.5M H_2SO_4	290	0.29	Appl. Catal. B, 2020, 279, 119394
18	Pd@Ir	0.1M $HClO_4$	245	3.33	Chem. Mater. 2019, 31, 5867-5875
19	Pd@Ir	0.1M $HClO_4$	300	1.01	ACS Catal., 2021, 11, 8239-8246
20	Ir-Co_3O_4	0.5M H_2SO_4	236	NA	Nat. Commun., 2022, 13, 7754

Table S6. Comparisons of HER activity of the as-prepared and commercial Ir/C and Pd/C catalysts.

Catalyst	Overpotentials (η) at 10 mA cm^{-2} (mV)	Tafel (mV dec^{-1})	HER-Mass activity (A mg_{Pd+Ir}^{-1}) at overpotential 20 (mV)	ECSA(m^2 g^{-1})
PdCu/C	40	48	0.39 (1.53 V)	52.9
PdCuIr/C	31	38	0.57 (1.53 V)	43.3
PdCuIrCo/C	19	28	1.6 (1.53 V)	49.1
Ir/C	41	51	0.4 (1.53 V)	28.9
Pd/C	60	106	0.36 (1.53 V)	60.6

Table S7. The HER performance of different noble metal based catalysts in acidic electrolyte solution.

No.	Catalyst	Electrolyte	Overpotential at 10 mA cm^2 (mV)	Mass activity (mA mg^{-1})	References
**1**	**PdCuIrCo/C**	**0.1 M $HClO_4$**	**19**	**1.06**	**This work**
2	IrCo PHNC	0.1 M $HClO_4$	21	NA	*Adv. Mater.* **2017**, *29*, 1703798
3	PdCu/Ir/C	0.1 M HClO4	20	1.90	Angew. Chem. Int. Ed. 2021, 60, 8243 – 8250
4	IrNi/C	0.1 M HClO4	25	1.64	*Small Methods* **2019**, *4*, 1900129
5	Ir/Si NW	0.05M H_2SO_4	22	NA	*Appl. Catal. B* ***2019**, 258, 117965*
6	IrFe/NC	0.05M H_2SO_4	22	1.83	Angew. Chem. Int. Ed. 2021, 60, 8243 – 8250
7	Ir-NR/C	0.05M H_2SO_4	28	3.9	*Appl. Catal. B* **2020**, *279*, 119394
8	IrNiCu HCSA	0.5M H_2SO_4	41	NA	*Sci. China Mater.* **2020**, *63*, 249
9	IrNiFe NPs	0.1M $HClO_4$	24	NA	*J. Mater. Chem. A* **2017**, *5*, 24836
10	Ir_6Ag_9 NTs	0.5M H_2SO_4	34	NA	*Nano Energy* **2019**, *56*, 330

Table S8. EXAFS data fitting results of Samples.

Sample	Path	CN^a	R(Å)^b	σ^2 (Å^2)^c	ΔE_0(eV)^d	R factor
Ir L3-edge (S_0^2=0.787)						
Ir_powder	Ir-Ir	12*	2.713±0.002	0.0032	9.4	0.0023
IrO2_powder	Ir-O	5.9±0.2	2.005±0.006	0.0032	2.7	0.0077
	Ir-Ir	1.9±0.4	3.179±0.029	0.0086	7.4	
	Ir-Ir	8.1±0.4	3.559±0.016		9.9	
Ir_PdCuIr	Ir-O	4.88±0.2	2.027±0.017	0.0021	9.0	0.0033
	Ir-Ir	2.5±0.3	2.719±0.019	0.0095	5.5	
Ir_PdCuIrCo	Ir-O	5.4±0.2	2.005±0.011	0.0012	3.1	0.0028
	Ir-Ir	0.8±0.3	2.719±0.011	0.0136	1.4	

^{a}*CN*, coordination number; ^{b}*R*, the distance between absorber and backscatter atoms; $^c\sigma^2$, the Debye Waller factor value; $^d\Delta E_0$, inner potential correction to account for the difference in the inner potential between the sample and the reference compound; *R* factor indicates the goodness of the fit. $S0^2$ was fixed to 0.838 and 0.787, according to the experimental EXAFS fit of Pd foil and Ir_powder by fixing *CN* as the known crystallographic value. * This value was fixed during EXAFS fitting, based on the known structure of Pd and Ir. Fitting conditions: *k* range：3.0 - 12.5; *R* range: 1.0-3.5 (Pd_PdCuIr and Pd_PdCuIrCo); *k* range：2.0 - 10.5; *R* range: 1.2-3.5 (Ir_PdCuIr and Ir_PdCuIrCo); fitting space: R space; *k*-weight = 3. A reasonable range of EXAFS fitting parameters: $0.800 < S_0^2 < 1.000$; $CN > 0$; $\sigma^2 > 0$ Å^2; $|\Delta E_0| < 10$ eV; *R* factor < 0.02.

Table S9. Ir *L3*-edge XANES Linear combination fitting (LCF).

Sample	Ir-powder	IrO_2-powder	*R* factor	*Reduced chi-square*
Ir-PdCuIr	0.216 (0.017)	0.784 (0.017)	0.0029	0.0010
Ir-PdCuIrCo	0.047 (0.022)	0.953 (0.026)	0.0046	0.0016

References

(1) Kresse, G.; Furthmüller, J. Efficiency of Ab-initio Total Energy Calculations for Metals and Semiconductors Using a Plane-Wave Basis Set. *Comp. Mater. Sci.* **1996**, 6, 15-50.

(2) Kresse, G.; Hafner, J. Ab initio Molecular Dynamics for Liquid Metals. *Phys. Rev. B* **1993**, 47, 558-561.

(3) Kresse, G.; Hafner, J. Ab initio Molecular-Dynamics Simulation of the Liquid-Metal–Amorphous-Semiconductor Transition in Germanium. *Phys. Rev. B* 1994, 49, 14251-14269.

(4) Kresse, G.; Furthmüller, J. Efficient Iterative Schemes for Ab Initio Total-Energy Calculations Using a Plane-Wave Basis Set. *Phys. Rev. B* **1996**, 54, 11169-11186.

(5) Perdew, J. P.; Burke, K.; Ernzerhof M. Generalized Gradient Approximation Made Simple. *Phys. Rev. Lett.* **1996**, 77, 3865-3868.

(6) Monkhorst, H. J.; Pack, J. D. Special Points for Brillouin-Zone Integrations. *Phys. Rev. B* **1976**, 13, 5188-5192.

(5) S.H. Talib, Z. Lu, X. Yu, K. Ahmad, B. Bashir, Z. Yang, J. Li, Theoretical inspection of M_1/PMA single-atom electrocatalyst: Ultra-high performance for water splitting (HER/OER) and oxygen reduction reactions (ORR), ACS Catal. **2021** 11, 8929-8941

(8) Jiao, M.; Song, W.; Li, K.; Wang, Y.; Wu Z. J. First-Principles Study on Nitrobenzene-Doped Graphene as a Metal-Free Electrocatalyst for Oxygen Reduction Reaction. *J. Phys. Chem. C* **2016**, 120, 8804−8812.

(9) Cox, J. D.; Wagman, D.D.; Medvedev, V.A.; *CODATA Key Values for Thermodynamics*. Hemisphere Publishing Corp., New York, **1984**, 1.

(10) S. Xue, G. Chen, F. Li, Y. Zhao, Q. Zeng, J. Peng, F. Shi, W. Zhang, Y. Wang, J. Wu, R. Che, *Small* **2021**, *17*, 2100559.

(11) Y. Xiong, H. Shan, Z. Zhou, Y. Yan, W. Chen, Y. Yang, Y. Liu, H. Tian, J. Wu, H. Zhang, D. Yang, *Small* **2017**, *13*, 1603423.

(12) M. J. Hÿtch, E. Snoeck, R. Kilaas, *Ultramicroscopy* **1998**, *74*, 131-146.

(13) Z. Xi, X. Cheng, Z. Gao, M. Wang, T. Cai, M. Muzzio, E. Davidson, O. Chen, Y. Jung, S. Sun, Y. Xu, X. Xia, *Nano Lett.* **2020**, *20*, 272.
